# Overlooked Sunfleck Properties and Potential Implications for Crop Improvement

**DOI:** 10.1111/gcb.71147

**Published:** 2026-10-06

**Authors:** Elias Kaiser, Rui Sun, Youngryel Ryu

**Affiliations:** ^1^ Research Institute of Agriculture and Life Sciences Seoul National University Seoul Republic of Korea; ^2^ Horticulture and Product Physiology, Plant Sciences Group Wageningen University & Research Wageningen the Netherlands; ^3^ Interdisciplinary Program in Agricultural and Forest Meteorology Seoul National University Seoul Republic of Korea; ^4^ Department of Landscape Architecture and Rural Systems Engineering Seoul National University Seoul Republic of Korea; ^5^ SNU Energy Initiatives Seoul National University Seoul Republic of Korea

**Keywords:** amplitude, cloudfleck, crop, duty ratio, fluctuating light, frequency, model, photosynthesis, sunfleck, windfleck

## Abstract

Sunflecks are ubiquitous in crop canopies, yet their properties are understudied. Recent data indicates that on sunny days, wind‐induced sunflecks within crop canopies (windflecks) occur roughly twice per second and at amplitudes of roughly 10% of full sunlight. However, studies on the effects of fluctuating light on photosynthesis and plant growth often use fluctuations that do not resemble sunflecks, possibly resulting in irrelevant crop improvement targets. In this review, first, we quantify this mismatch between experiments and reality, showing that most studies measuring light intensities in crop canopies did so too infrequently to capture all sunfleck properties, while studies either simulating dynamic photosynthesis or growing plants under fluctuating light did so using much slower fluctuations than those often encountered in the field. Second, we attempted to estimate the losses of photosynthesis due to sunflecks: using light intensity fluctuations recorded inside a rice canopy at high frequency (100 Hz) on a sunny day, a dynamic photosynthesis model based analysis calculated minimal losses (0.85%–1.24% diurnally; relative to steady‐state simulations), suggesting that sunflecks are too frequent, short, and weak to cause substantial losses due to slow dynamics of photosynthesis. We found that the choice of light intensity pattern matters enormously, as estimated losses reached 13% diurnally when using a less realistic light intensity pattern. We also introduce a framework for determining photosynthesis under windflecks based on pulsed light experiments. Thirdly, we present an overview of effects of global climate change on dynamic photosynthesis and crop growth under fluctuating light, concluding that likely, rising [CO_2_] will benefit photosynthesis under sunflecks, whereas heat, VPD and drought may be detrimental. Finally, we suggest future research directions, noting that new targets for crop improvements under sunflecks will require a much better characterization of sunflecks, their windfleck and cloudfleck components, and their effects on photosynthesis and plant growth.

## Graphical abstract

Crop canopies experience frequent, high‐frequency “windflecks” (~1–2 Hz, ~10% amplitude), yet most experiments and models use ~500‐fold slower fluctuations. Using realistic field data, we estimate that diurnal photosynthesis losses under sunflecks are minimal (~1%), not the ~13%–21% assumed from unrealistic light patterns—reshaping targets for crop improvement under fluctuating light.

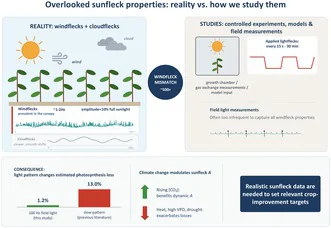

## 1 Introduction

Almost six decades ago, Keith McCree and Bob Loomis, two pioneers in photosynthesis research, published an article simply and aptly named “Photosynthesis in fluctuating light,” in which they exposed cucumber plants to a range of light intensity amplitudes and fluctuations that they considered close to natural (up to 71 light intensity changes s^−1^, i.e., up to 71 Hz) and measured their photosynthetic output (McCree and Loomis 1969). They concluded “that steady‐state photosynthetic rates may safely be used to compute the rates in fluctuating light” (McCree and Loomis 1969). Today, this conclusion reads nothing short of revolutionary, as it implies that under the rapid light intensity fluctuations found in nature, net photosynthesis rate (*A*) reacts instantaneously, and therefore no reduction compared to steady‐state responses to different light intensities should be expected. Despite this finding, the article was mostly ignored by the photosynthesis community (< 100 citations in Google Scholar to date), which broadly went on to make the opposite claim: a large body of literature now suggests that *A* reacts slowly to sudden increases in natural sunlight above a low light intensity baseline value (sunflecks), and that we can “speed up” this response to improve crop growth in the field.

In the 80's and 90's, major insights on *A* under fluctuating light (termed dynamic *A*, as opposed to steady‐state *A* under constant light intensity) were driven by research on plants in the understory of forests, particularly by the pioneering work of Robert W. Pearcy (Pearcy et al. 1996, 1997; Sassenrath‐Cole and Pearcy 1992; Valladares et al. 1997). Although Pearcy and colleagues also conducted highly relevant work on field crops, particularly soybean (Pearcy and Seemann 1990; Pons et al. 1992; Pons and Pearcy 1992), this was not yet driven by the current focus on crop improvement. This focus was broadly introduced by seminal research of Stephen P. Long's team a decade ago, which showed that by speeding up the relaxation of photoprotection following sunflecks, tobacco plants in the field showed increased growth (Kromdijk et al. 2016). Since then, the number of studies mentioning photosynthesis under fluctuating light has increased exponentially, even when normalized by all studies mentioning photosynthesis (Figure S1). This suggests substantial interest in the research community in this topic, especially with regards to crop improvement under sunflecks.

In the face of human‐made climate change, continued increases in population size, and diminishing arable land, the search for crops with enhanced yields and improved resource use efficiency is more pressing than ever. A likely candidate process to improve is *A*, as this is well below its theoretical maximum (Zhu et al. 2010), and several promising avenues to improve it are currently being explored (Croce et al. 2024; Long et al. 2025), including its response to fluctuating light intensity (Kaiser et al. 2019; Long et al. 2022). However, as we will argue in this article, previous approaches have often overlooked the relevant frequencies and other properties of sunflecks in the field, and this may lead to unrealistic targets for improvement.

Photosynthesis drives plant growth, and in the field often occurs under turbulent light intensities (Figure 1), which are caused by wind‐induced leaf and cloud movement. In fact, it is now well documented that in the middle of a sunny day, sunflecks inside crop canopies are extremely plentiful and short, as they occur more than once per second on average (Burgess et al. 2021; Durand et al. 2021, 2026; Durand and Robson 2023). However, this can only appreciably be quantified when light intensity is logged at rates of ~100 Hz (Durand et al. 2021). Also, on cloudy or overcast days, there may be many fewer short sunflecks (Figure 1e,f), but a detailed analysis of weather effects on these short fluctuations is currently lacking.

**FIGURE 1:**
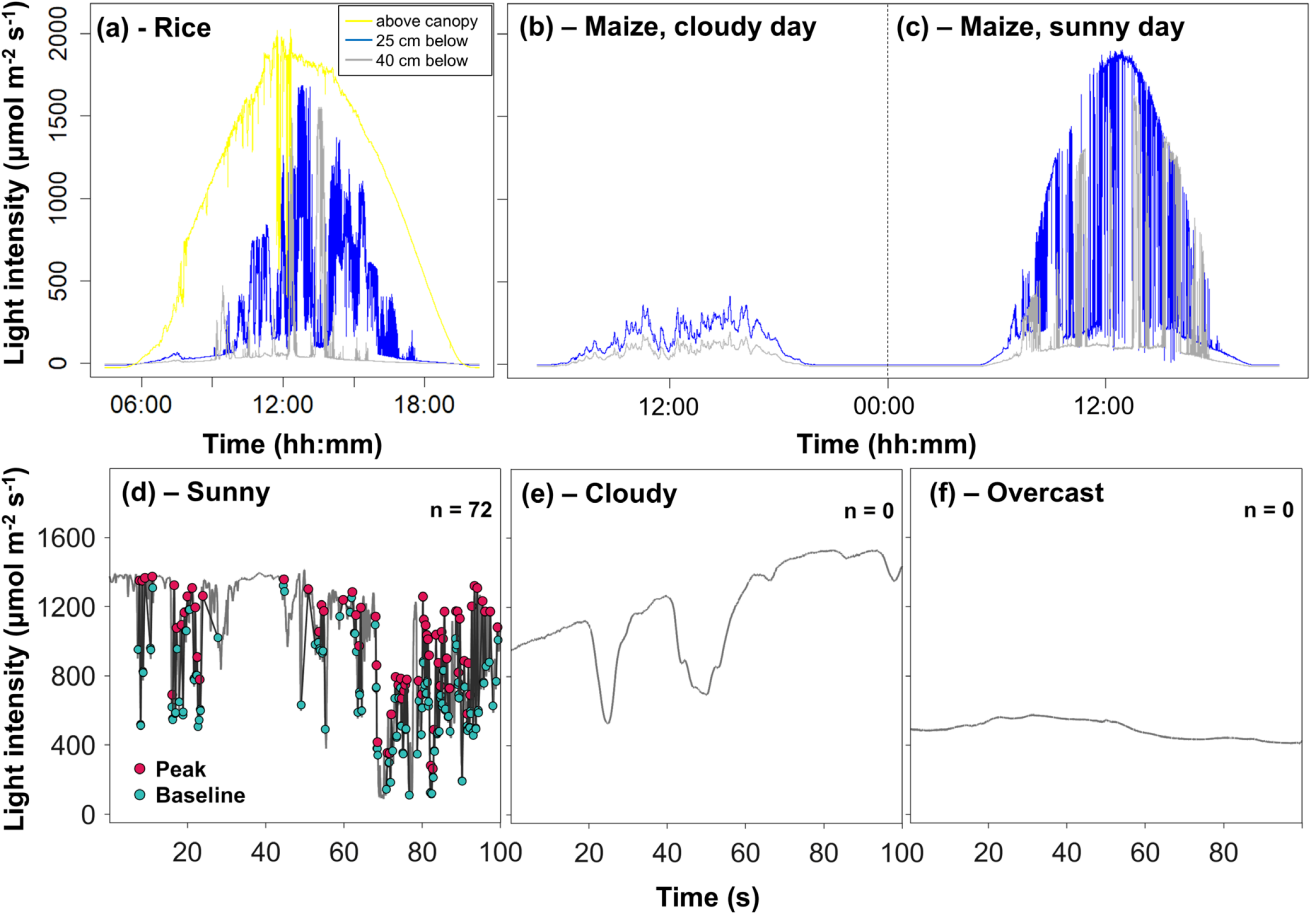
Light intensities measured in rice and maize canopies during the summer in South Korea, and application of a sunfleck identification method under various weather types. (a–c) Measurements were conducted above the canopy (yellow line), 25 (rice) or 40 cm (maize) beneath the top of the canopy (blue lines), as well as 50 (rice) or 90 cm (maize) beneath the top of the canopy (gray lines). (d–f) Application of the method by Durand et al. (2021) to identify sunflecks within 100‐s time frames during a sunny (d), cloudy (e) and an overcast day (f); *n* denotes the number of identified sunflecks; for complete overview see Figure S2. Rice data were logged on 19–30 July 2025 near Cheorwon. Maize data were logged on 22 and 23 June 2025 in Suwon. All data were logged at 100 Hz, using Li‐190R quantum sensors (Li‐Cor Biosciences, Lincoln, NE, USA) connected to dataloggers (DI‐2008, DATAQ, Akron, Ohio, USA).

The response of *A* to sunflecks is typically probed at frequencies that are much lower than those encountered in nature. An example of this is the measurement of photosynthetic induction, where leaves that had been adapted to a stable low light intensity for several tens of minutes are exposed to a high (near‐) saturating light intensity until reaching a steady state (for review see Acevedo‐Siaca and McAusland 2025). Under such conditions, *A* takes a few to several tens of minutes to transition from the initial (low‐light) to the final steady‐state rate. Measuring photosynthetic induction is undoubtedly useful for comparing the effects of various treatments and genotypes on dynamic *A* (Kaiser, Kromdijk, et al. 2017; Kaiser, Zhou, et al. 2017; Shao et al. 2024; Zhang et al. 2018, 2022, 2024) or for calibrating models of dynamic *A* (Joubert et al. 2023; Morales et al. 2018). However, the use of photosynthetic induction can be misleading when thinking about crop canopies in the field, as (1) light intensities anywhere in the canopy are typically too unstable for leaves to reach any true steady state, and (2) a large fraction of *A* takes place in the top canopy layer, which rarely ever faces deep shade, unlike lower‐canopy leaves. Furthermore, whether fast photosynthetic induction in a given genotype correlates with faster crop growth under sunflecks is entirely unclear.

The “slow view” of dynamic *A* as informed by measurements of photosynthetic induction has also percolated to experiments of plant growth under controlled fluctuations in light intensity, and to models of dynamic *A*. For example, there is a much‐repeated claim that due to slow kinetics, diurnal *A* in the field is reduced by up to 21% (Taylor and Long 2017). However, this claim is based on light fluctuations that were applied at most once per minute in a mathematical model of dynamic *A* (Taylor and Long 2017), and thus much more slowly than natural sunflecks, at least under sunny conditions (Figure 1d). Sunflecks can be defined as a mixture of wind‐ and cloudflecks (Table 1), assuming that the Earth's rotation and the resulting “solar movement” result in negligibly few additional fluctuations. Windflecks are rapid (~< 1 s) and are generated inside the canopy due to wind‐induced plant and leaf movement (Burgess et al. 2021; Durand and Robson 2023; Durand et al. 2026). Cloudflecks, on the other hand, last 3–7 min on average, are interrupted by similarly long gaps as their duration, and are comparably few (16–50 per day; Kaiser et al. 2018; Knapp and Smith 1988; Mol et al. 2023; Sellaro et al. 2025). In this review, we refer to the general phenomenon of natural light intensity fluctuations inside crop canopies as sunflecks, and only refer to them as wind‐ or cloudflecks when either short, wind‐driven fluctuations or long, cloud‐driven fluctuations are meant. We broadly argue that the effects of windflecks on plant physiology and growth have been ignored, whereas the effects of cloudflecks may have been overstated. In other words, there seems to be a mismatch between reality—very fast and plentiful sunflecks in the field (Figure 1, Table S1)—and experimental and modelling reproductions (“lightflecks,” Table 1) that claim to characterize leaf or plant responses to sunflecks, but generally have very different properties. Here, we (1) review the literature to quantify this mismatch, (2) highlight the possible implications of this mismatch for crop improvement, (3) estimate the reductions in *A* that may occur under sunflecks, (4) summarize the known effects of global climate change on crop *A* and growth under fluctuating light, and (5) provide several research targets that may narrow the gap between lab and field.

**TABLE 1:** Classification of various types of fluctuating light.

Name	Description	References
Sunfleck	Transient increase in natural light intensity above a defined threshold. Sunflecks can occur above or within plant canopies, and may be caused by movement of clouds, plants, or single leaves	Kaiser et al. (2018)
Cloudfleck	Fluctuation in light intensity above the canopy due to clouds. Typically lasts several minutes. Subtype of sunfleck	Kaiser et al. (2018)
Windfleck	Transient, sub‐second increase in natural light intensity above a defined threshold, as measured inside plant canopies and caused by wind‐induced plant swaying and leaf movement. Subtype of sunfleck	Burgess et al. (2021); Durand et al. (2026); Durand and Robson (2023)
Shadefleck	Transient reduction in natural light intensity below a defined threshold. Mirror image of sunfleck	Kaiser et al. (2018); van Westreenen et al. (2026)
Lightfleck	Artificial fluctuation in light intensity, made to study plant responses to fluctuating light	Pearcy et al. (1996)

*Note:* Descriptions are based on prior literature and are synthesized to the best of our knowledge. However, we acknowledge that for some terms, conflicting definitions exist (e.g., Sellaro et al. 2025; Smith and Berry 2013).

## 2 Crops, Fluctuating Light, and the Mismatch Between Experiments and Reality

To assess the mismatch between sunflecks and their experimental representations, we reviewed the literature (Data S1) with regards to three types of studies (Figure 2): Firstly, we collected studies that measured light intensities inside crop canopies (21 studies; Table S2), focusing on the rates and durations at which these were logged. Secondly, we looked at all studies that used mathematical models to simulate the dynamic response of *A* to fluctuating light (29 studies; Table S3), asking at which frequencies light intensity was changed to trigger responses in calculated *A*. Thirdly, we assessed all studies (31 studies)—and all experiments within these studies (90 experiments; Table S4)—in which plants were grown under lightflecks, typically with the aim of comparing these plants to those grown under a constant light intensity with the same average light intensity and photoperiod. Also, we reviewed the literature with regards to windfleck frequencies, duration and amplitude in crop canopies (Figure 3, Table S1). These data had been obtained in canopies of oat ( *Avena sativa* ), barley ( *Hordeum vulgare* ), broad bean ( *Vicia faba* ), rapeseed ( *Brassica napus* ) and 10 cultivars of winter wheat ( *Triticum aestivum* ) in field trials in Finland and the UK (Durand et al. 2021, 2026; Durand and Robson 2023).

**FIGURE 2:**
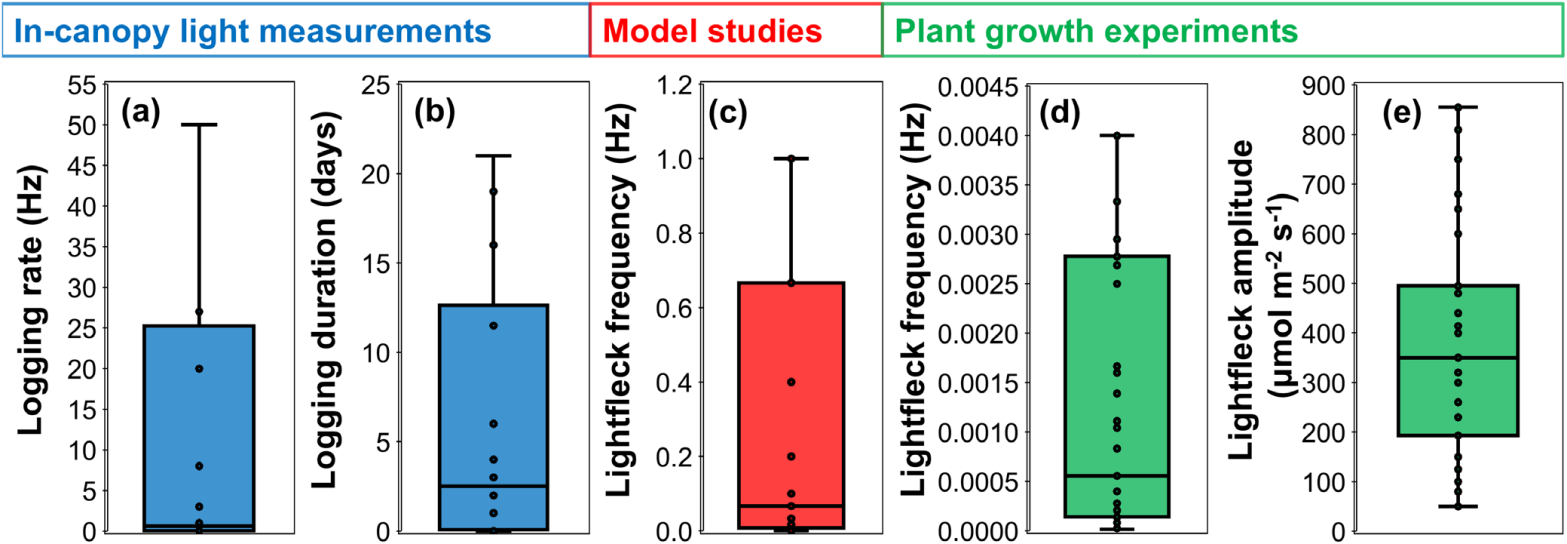
Overview of studies characterizing sunflecks and their effects on photosynthesis as well as plant growth. (a) Rate of logging of light intensities as measured inside crop canopies (*n* = 15). (b) Total duration of light intensity recordings per study (*n* = 19). (c) Highest frequency of lightflecks applied in studies of dynamic photosynthesis models (*n* = 21). (d) Lightfleck frequency applied in experiments designed to grow plants under controlled light intensity fluctuations (*n* = 87). (e) Amplitude of lightflecks in fluctuating light growth experiments (*n* = 84). Boxplots show 25th and 75th quantile, the line inside boxes depicts the median, and circles show single data values. Outlier datasets were excluded: three in (a), two in (b), four in (c), three in (d), and four in (e).

**FIGURE 3:**
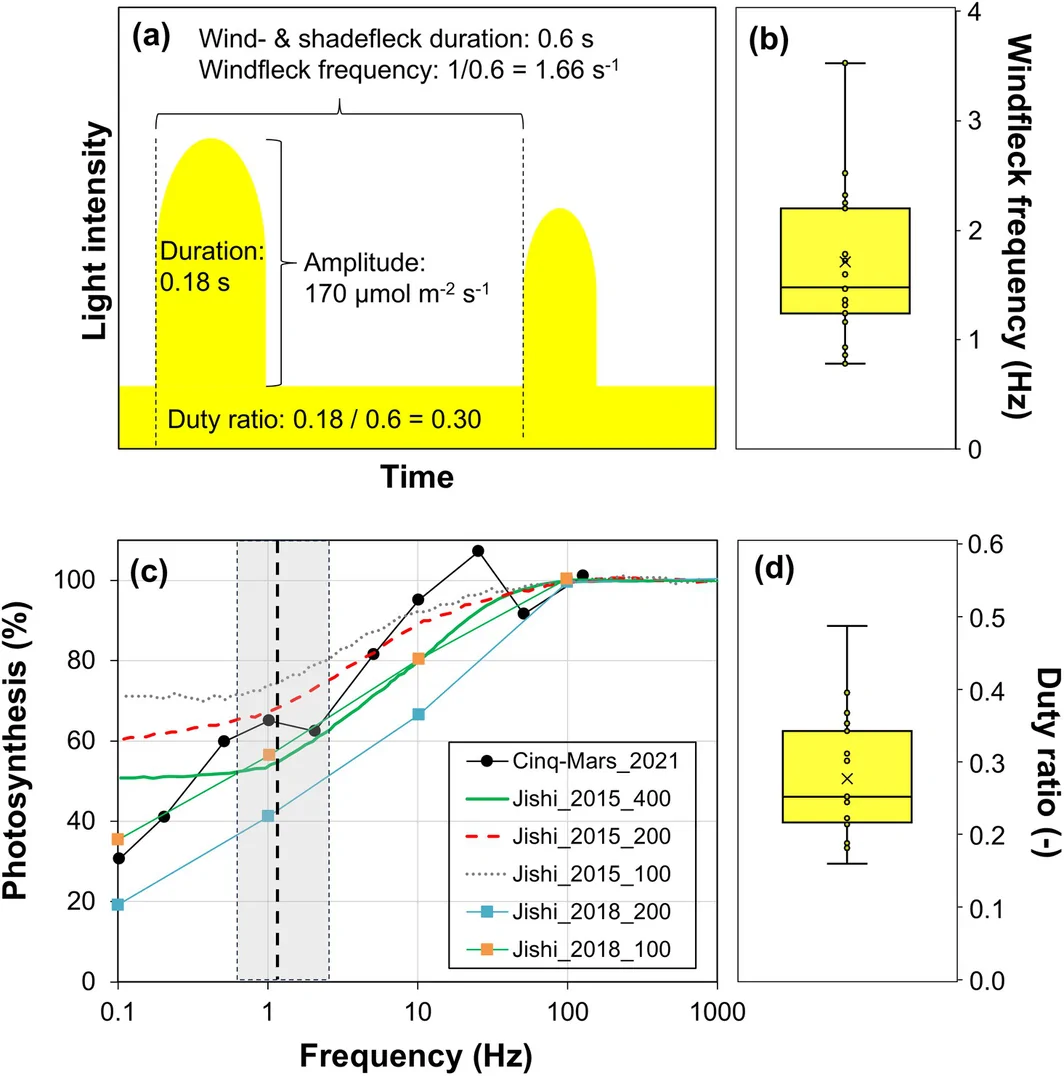
Windfleck frequency and duty ratio and its potential effects on photosynthesis. (a) Schematic to explain the meaning of windfleck frequency (1/combined duration of wind‐ & shadefleck) and of windfleck duty ratio (duration of windfleck/combined duration of wind‐ & shadefleck). (b) Windfleck frequency. (c) Photosynthesis as a function of frequency of pulsed light at a duty ratio of 0.25. Photosynthesis rates were normalized by rates recorded in the 100–1000 Hz range. Data digitized from Cinq‐Mars and Samson (2021), Jishi et al. (2015), and Jishi et al. (2018); the latter two papers present data at several average light intensities (100–400 μmol m^−2^ s^−1^); these are denoted by the last number shown in the legend. Gray shaded area and striped vertical line represent range and average of windfleck frequency. (d) Windfleck duty ratio. Boxplots show 25th and 75th quantile, the line inside boxes depicts the median, “x” depicts the average, and circles show single data values. Data in (a), (b), and (d), and gray shaded area in (c), are based on data collected by Durand et al. (2021, 2026), Durand and Robson (2023) in crop canopies under full sunlight; these are shown in Table S1 (*n* = 17).

We found that most studies logged light intensities at very low frequencies, resulting in a median 0.2 Hz (Figure 2a), and thus a 500‐fold difference from the 100 Hz logging rate recommended by Durand et al. (2021). Further, light intensities were typically logged for short durations only, at a median 2.5 days per study (Figure 2b), suggesting that the effects of changes in leaf area index (LAI) or weather were generally not taken into account. Crucially, not a single study logged light intensities at sufficiently high frequencies for several days in a row, such that assessing the effects of different climatic conditions on sunfleck properties was not possible (although Nishimura et al. (1998) logged at 50 Hz for 4 days, and Tong and Hipps (1996) logged at 27 Hz for 21 days). Furthermore, only 13 of 20 studies attempted to quantify any properties of these fluctuations (Table S2).

Among the 25 modelling studies of dynamic *A*, the fastest simulated lightfleck per study was a median 0.07 Hz (i.e., once every 15 s; Figure 2c), and thus much slower than the average 1.66 Hz of windflecks (Figure 3b). Furthermore, only 5 of 25 modelling studies attempted to model the dynamic behavior of *A* under light intensity patterns that mimicked those of field conditions (Table S3). Only 4 out of 25 studies modelled *A* under fluctuations faster than 1 Hz (Jishi et al. 2015; Morales et al. 2018; Porcar‐Castell et al. 2006; Roden 2003), meaning that most models were not tested under the frequencies relevant to windflecks. Furthermore, the four models simulating *A* at ≥ 1 Hz were validated by measurements of *A* at lower frequencies only, so it is unclear whether their predictions at these higher frequencies are accurate. Finally and most strikingly, the lightfleck frequency among plant growth experiments was even lower than that found in modelling studies, namely a median 0.00056 Hz (once every thirty minutes) among 87 experiments (Figure 2d). The median lightfleck amplitude was 350 μmol m^−2^ s^−1^ (Figure 2e), which is ca. twice the average amplitude found in windflecks (170 μmol m^−2^ s^−1^; Figure S3).

In conclusion, the vast majority of studies looking at photosynthesis and/or growth under fluctuating light have so far used unrealistic frequencies to characterize the effects of windflecks: researchers often did not log light intensities frequently or long enough to properly characterize them, and used lightflecks that were far too slow to realistically assess the effects of windflecks on (simulated) *A* or growth. The chosen lightflecks are closer to cloudflecks in their duration (3–7 min), but given the relative paucity of cloudflecks (only 16–50 per day), their potential effects on diurnal canopy photosynthesis may have been overstated.

## 3 Sunfleck Effects on Leaf Photosynthesis and Crop Growth

### 3.1 Physiology of Leaf *A* Under Fluctuating Light

As the photosynthetic machinery deactivates slowly when plunged from high light into shade, and then again activates slowly upon sunfleck exposure, using very slow light intensity fluctuations likely inflates estimates of foregone *A* under sunflecks. The key processes underlying dynamic *A*, ranked from fastest to slowest, are: (1) the change in the size of metabolite pools of the Calvin Benson Bassham cycle (CBB), (2) induction and relaxation of non‐photochemical quenching (NPQ), (3) activation states of key enzymes in ribulose‐1,5‐bisphosphate (RuBP) regeneration, (4) the activation state of ribulose‐1,5 bisphosphate carboxylase oxygenase (Rubisco), (5) transient changes in mesophyll conductance (*g*_m_) and (6) stomatal opening and closure. These processes react within seconds (CBB metabolite pools) to tens of minutes (stomatal conductance; *g*_s_) to light intensity changes (Table 2; Kaiser et al. 2015, 2018; Liu et al. 2021; Woning et al. 2025). In the context of windflecks, only some of these processes may matter, as leaves may hardly ever be in the shade for long enough for slower processes to change their activation state.

**TABLE 2:** Time constants of key processes in dynamic photosynthesis, after in‐ and decreases in light intensity.

Process	Time constant (min)						References
	Increase			Decrease			
	Avg.	Min.	Max.	Avg.	Min.	Max.	
CBB metabolite pool size	0.10			0.55			Sassenrath‐Cole and Pearcy (1992); Xu et al. (2025)
Non‐photochemical quenching	1.7	1.2	2.2	0.4	0.2	0.7	Ferguson et al. (2025); Sahay et al. (2023, 2024); Vath et al. (2026)
RuBP regeneration	2.4	0.8	3.6	2.5	1.4	4.0	Kaiser et al. (2018)
Rubisco activation state	3.8	2.9	5.0	10.8	1.0	28.9	Seemann et al. (1988); Taylor et al. (2022); Woodrow and Mott (1989)
Mesophyll conductance	5.9	2.9	8.6				Liu et al. (2021); Pan et al. (2025); Sakoda et al. (2021)
Stomatal conductance	21.8	1.1	85.2	15.4	0.7	106.2	Woning et al. (2025)

*Note:* Averages, minimum, and maximum values are displayed if available. Numbers for Calvin Benson Bassham cycle (CBB) intermediate pools represent those of C_3_ plants only.

Because they have been reviewed in great detail elsewhere (e.g., Kaiser et al. 2015, 2018, 2019; Lawson and Blatt 2014; Long et al. 2022; Pearcy et al. 1996), here we will provide only a brief overview of the processes underlying dynamic *A*. In a leaf in the shade that is suddenly exposed to a sunfleck, the increase in light energy absorbed by the chlorophylls and other accessory pigments leads to a sudden increase in the rate of water splitting at photosystem II (PSII) and consequent linear electron transport along the thylakoid membrane between PSII, the cytochrome b_6_f complex, and photosystem I (PSI), leading to the reduction of NADP^+^ to NAPDH. Light‐energy driven electron transport also leads to protons being enriched in the chloroplast lumen relative to the chloroplast stroma; this difference powers the ATP synthase and thus the production of ATP, reduces the electron transport conductance of the cytochrome b_6_f complex (photosynthetic control), and triggers the formation of photoprotective NPQ. NADPH and ATP are used in the CBB to generate various forms of precursor for the CO_2_ (and O_2_) acceptor molecule, RuBP; hence, the pool sizes of these various metabolites, as well as the turnover rate of the CBB, increase. The activation state of several enzymes that control the rate of RuBP regeneration also quickly increases. The activation state of the enzyme that is key to fixing CO_2_, Rubisco, also increases, but at a slower pace. At an even slower rate, the ease with which CO_2_ diffuses from outside the leaf toward Rubisco increases, as the stomata open, and as *g*_m_ increases. If the sunfleck persists for long enough, these processes reach a steady state, allowing *A* to reach a stable, maximum rate under the given environmental conditions. When the sunfleck ends and the leaf is plunged back into shade, linear electron transport slows down quickly. However, the pools of ATP, NADPH and CBB intermediates allow for some residual *A*, a phenomenon lasting a few seconds, and known as post‐illumination CO_2_ gain. Within tens of seconds after the reduction in light intensity, some photorespiratory intermediates are rapidly respired, leading to a transient dip in *A*, called post‐illumination CO_2_ burst. Further, NPQ relaxes quickly, but is transiently large enough to compete for excitation energy with linear electron transport and thus reducing *A* under low light intensity. The activation state of the enzymes controlling RuBP regeneration in the CBB is reduced within minutes, as is that of Rubisco. Finally, stomata close and *g*_m_ is reduced.

It could be hypothesized that the more slowly a given process deactivates or relaxes when a change from high to low light intensity has occurred (e.g., stomatal closure; Table 2; Lawson and Blatt 2014), the less likely it is to limit dynamic *A* in the field, assuming that windflecks dominate over cloudflecks in terms of their relative effects on dynamic *A*. Hence, changing these slowly deactivating processes may not lead to crop improvements in the field. In fact, so far the only crops showing improved growth under sunflecks were those with faster NPQ relaxation (De Souza et al. 2022; Kromdijk et al. 2016), which happens comparably quickly (Table 2): changes in the rate of NPQ relaxation might have an immediate effect on photosynthetic efficiency directly after the transition from a higher to a lower light intensity. Even if the effect may be minuscule per sunfleck, when integrated over countless sunflecks throughout a growing season it may become substantial. However, the relative importance of wind‐ and cloudflecks on dynamic *A* in crop canopies has not been elucidated.

### 3.2 Toward Quantifying Sunfleck‐Induced Losses in Photosynthesis

We estimated the losses in *A* that might occur under sunflecks through a dynamic *A* model that was applied to several light intensity patterns (Figure 4). Further, we explored the use of a new framework to quantify losses in *A* under windflecks, using reported windfleck properties and measured *A* under pulsed light (Figure 3).

**FIGURE 4:**
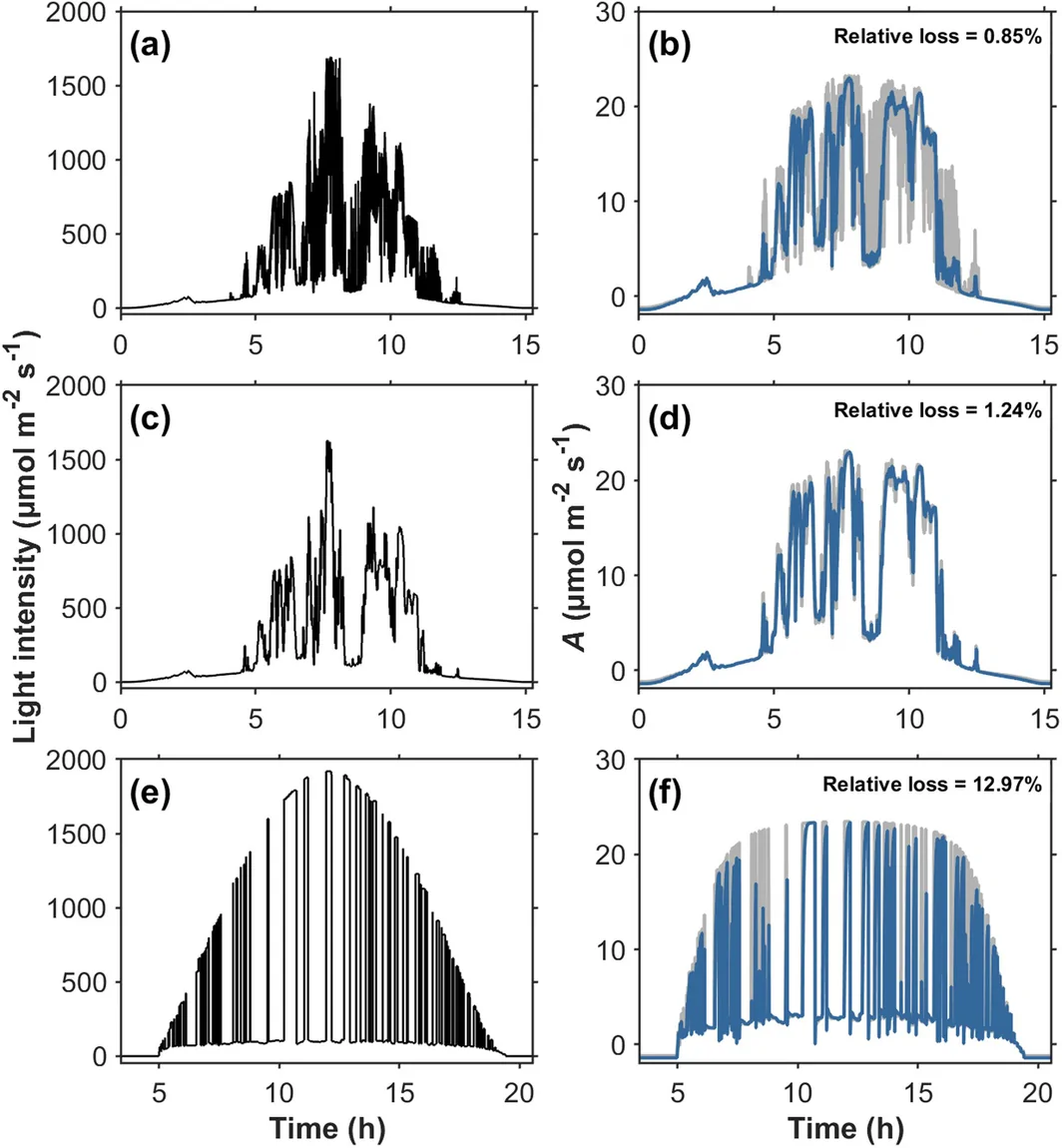
Model analysis of dynamic and steady‐state *A* under fluctuating light. (a, c, e) Light intensity patterns during the photoperiod. (b, d, f) Simulated *A* using a dynamic (blue lines) and a steady‐state model (gray lines). The relative loss was calculated as the percentage reduction in cumulative dynamic *A* relative to the steady‐state simulation. In Panels (a–d), light intensities recorded in a rice canopy grown in Cheorwon, South Korea in the summer of 2025 were used. In Panels (e) and (f), the light intensity pattern by Taylor and Long (Taylor and Long 2017) was used. In Panels (a) and (b), light intensity was updated every 0.01 s (100 Hz), whereas in (c–f), it was updated once per minute (0.0166 Hz). For details, see Data S2.

#### 3.2.1 Photosynthesis Model Based Analysis

For the model analysis, the dynamic leaf *A* model by Kirschbaum et al. (1998) was used. This model accounts for dynamic changes in photosynthetic intermediate pool sizes, the activation states of RuBP regeneration and Rubisco, as well as *g*_s_. Some modifications were introduced here to simplify the *g*_s_ module (see Data S2). The model was parameterized for rice leaves using data by Yamori et al. (2020) and other sources (Table S5). We found that the model simulated steady‐state and dynamic *A* and *g*_s_ responses to environmental perturbations realistically (Figure S4). We then used the original (dynamic) version of the model, as well as a steady‐state version that responds instantaneously to any changes in light intensity, to estimate the relative loss in diurnal *A* due to slow responses by *A* to fluctuating light. When conducting this analysis on light intensity data that had been recorded at 100 Hz inside a rice canopy during a summer day (Figure 4a), we found the loss in dynamic *A*, relative to steady‐state *A*, to be negligible (0.85%, Figure 4b). A sensitivity analysis of key transient limitations suggested Rubisco activation state to have the strongest effects, followed by RuBP regeneration activation state, while rates of stomatal opening and closure had comparably small effects (Table S6). To test the extent to which slower recording of light intensity data in the field would affect estimated *A*, we downsampled the original light intensity data to one datapoint per minute (0.0166 Hz; Figure 4c), and found that the relative loss in dynamic *A* was higher (1.24%, Figure 4d), although not by much. Also, when using 1 min averages of the 100 Hz data rather than one single datapoint per minute, the relative loss in dynamic *A* was similar (0.99%; data not shown). It thus seems that in this example at least, a lower rate of light intensity recordings would not have changed our conclusions much.

As the very small differences between dynamic and steady‐state *A* (0.85%–1.24%) stand in stark contrast to prior modelling results, we also ran our model analysis on the light intensity pattern used by Taylor and Long (2017), who had reported a relative loss of 21% of diurnal dynamic *A* relative to steady‐state *A*. Using that pattern (Figure 4e) at 1 min resolution (same resolution as in Figure 4c,d), we obtained a relative loss of ca. 13%, and thus a much larger relative loss than when using the light intensity pattern recorded in rice (Figure 4b,d). The difference to the 21% relative loss estimated by Taylor and Long (2017) may be due to their use of a larger time constant for Rubisco activation (*𝜏*_i,R_: 180 s) and a smaller time constant for Rubisco deactivation (*𝜏*_d,R_: 300 s) in wheat, than in our model formulation for rice (*𝜏*_i,R_: 161 s, *𝜏*_d,R_: 450 s; Table S5). Regardless, this analysis highlights the immense importance of choice of light intensity pattern for estimating relative losses in dynamic *A*: light intensity data as recorded in a rice canopy (even when downsampled to 1 min, Figure 4c) resulted in almost no estimated losses (1.24%, Figure 4d), whereas the light intensity pattern with long low light intervals throughout the day (Figure 4e) resulted in significant estimated losses (12.97%, Figure 4f).

One caveat of our model is that it does not include dynamic changes in *g*_m_ and NPQ. Given the slow kinetics of *g*_m_ during photosynthetic induction (Table 2), we doubt that its inclusion would have changed the results much, but we also note that the rate of “*g*_m_ deactivation” after light‐shade transitions is unknown (Table 2). Including NPQ kinetics, on the other hand, would have led to stronger post‐illumination losses in dynamic *A*, as NPQ reacts quickly to changes in light intensity (Table 2) and has been proven to dynamically limit *A* after light‐shade transitions (Kromdijk et al. 2016). However, to what extent this adds limitations during light intensity fluctuations as rapid as shown here (Figure 4a) needs to be explored; to our knowledge, the effects of dynamic NPQ on sunfleck *A* have only been explored under slower fluctuations (Morales et al. 2018; Wang et al. 2020). Either way, we hypothesize that even if NPQ and *g*_m_ had been included in the model, the main conclusion (namely that the choice of light pattern strongly affects estimations of diurnal foregone *A*) would still hold.

#### 3.2.2 Theoretical Framework for Estimating “Windfleck *A*”

It might also be possible to estimate the reduction of *A* under windflecks from measurements of *A* under artificially pulsed light, where short square‐wave light pulses are interspersed by dark periods. Unfortunately, only few such studies exist. Important properties of pulsed light include its frequency, duty ratio (the duration of the light phase relative to the total duration of light and dark phase; Figure 3a), background light intensity, and amplitude. Generally, as the frequency of light pulses increases, time‐integrated *A* approaches that of steady‐state *A* under the same average light intensity, but never surpasses it (Sager and Giger 1980). At very high frequencies (100–1000 Hz), time‐integrated *A* is identical to steady‐state *A*, independently of duty ratio (Cinq‐Mars and Samson 2021; Jishi et al. 2015, 2018). At such high frequencies, the quantum yield of regulated NPQ was lower than at lower frequencies (Cinq‐Mars and Samson 2021), suggesting that pulsed light of high frequencies is less stressful than that of lower frequencies. It seems that at these high frequencies, interruptions to light energy supply are so short that they cause only minor fluctuations in linear electron transport and downstream processes, and consequently the photosynthetic machinery can integrate all incoming light energy.

In the 0.8–3.5 Hz frequency range at which windflecks occur (Figure 3b, Table S1), *A* under pulsed light tends to be lower than steady‐state *A* (Cinq‐Mars and Samson 2021; Tennessen et al. 1995). However, the extent of its reduction depends strongly on duty ratio (stronger reduction as duty ratio declines) as well as light intensity (stronger reduction as light intensity increases; Jishi et al. 2015, 2018). For example, Jishi et al. (2015) showed that in lettuce leaves measured at a duty ratio of 0.5, *A* declined by < 15% in the 0.1–10 Hz range compared to *A* at 100–1000 Hz, whereas at a duty ratio of 0.25, reductions in *A* at lower frequencies approached 50%. Hence, for quantifying the effects of fluctuating light on time‐integrated carbon gain, its frequency, duty ratio and average light intensity matter enormously.

Defining a “duty ratio” for sunflecks is not straightforward (Figure 3a), as this is usually calculated using artificial, clear (often square‐wave) and repeated light patterns, not the messy patterns encountered in nature. Nevertheless, based on high‐frequency light intensity measurements in fully sunlit crop canopies (Durand et al. 2021, 2026; Durand and Robson 2023), we calculated an average “duty ratio” of 0.276 (±0.087 SD, *n* = 17; Figure 3d). Hence under full sunlight, windflecks occur roughly 25%–30% of the time. This number is close to a duty ratio of 0.25, which was used by Cinq‐Mars and Samson (2021) and Jishi et al. (2015, 2018) to estimate *A* under pulsed light. When combining their results, the mean reduction in *A* (compared to that at frequencies > 100 Hz, which is equivalent to steady‐state *A*) was close to 40% (Figure 3c). However, this is likely a huge overestimation of lost *A* under windflecks, as these have relatively small amplitudes (170 μmol m^−2^ s^−1^ on average; Figure S3), and occur under a substantial background light intensity. On the other hand, data shown in Figure 3c was obtained using light pulse intensities of 400–2300 μmol m^−2^ s^−1^ that were interrupted by darkness (Cinq‐Mars and Samson 2021; Jishi et al. 2015, 2018), and thus under much more extreme conditions than occur in the field. Further, (i) the large variability between datasets shown in Figure 3c precludes more precise quantifications of lost *A*, (ii) data in Figure 3c are based on measurements from two species only (lettuce and sunflower) that were grown in climate chambers, (iii) windfleck properties (Table S1) were only measured under full sunlight, not a wider range of weather conditions, and (iv) in full crop canopies in the field, the situation may be even more complex (see below). Furthermore, windflecks tend to appear in irregular clusters that are caused by wind gusts (e.g., Durand and Robson 2023). Sunfleck clustering would add second‐order fluctuations, and would complicate the analysis further. Altogether, these results should thus be interpreted with much caution. In our opinion, the estimated loss in *A* based on this analysis (~40% loss, Figure 3c) is unrealistically high (due to large pulse light amplitudes and darkness between pulses, as discussed above), but the above framework may nevertheless be used to better quantify *A* under windflecks.

### 3.3 Crop Growth Under Fluctuating Light

Plant growth is the result of many processes that interact across time and space. For example, *A* by all CO_2_‐fixing organs (leaves, stems, fruits) over the entire growing season produces the carbohydrates that are used in growth and maintenance respiration that is required for crop growth. For dynamic *A*, the time component is relevant as throughout the growing season, the level of self‐shading increases with LAI. Furthermore, different weather conditions strongly influence sunflecks, as light intensity fluctuations were found to be suppressed under overcast compared to sunny conditions (Figure 1, Nishimura et al. 1998); however, a full quantitative exploration of weather effects (e.g., cloudiness, direct: diffuse ratio) on sunfleck properties does not yet exist, nor is there an extended analysis that separates wind‐ from cloudflecks. As for the space component, the uppermost canopy layer intercepts most incoming light, which reduces available light with depth exponentially. It could be hypothesized that because there is no self‐shading, the upper leaf layer experiences fewer and shallower sunflecks, but this remains to be shown. Known effects of crop canopy architecture on sunfleck properties (Burgess et al. 2021; Durand et al. 2021, 2026) can be summarized as: in taller canopies, windflecks tended to be shorter, occur at a lower frequency and with longer low light intervals. A larger plant area index correlated with lower windfleck intensity and with fewer long sunflecks (> 10 min). In wheat, a larger leaf‐to‐tiller mass correlated with shorter windflecks. A more open plant structure was related to higher‐frequency windflecks that occurred at shorter intervals. A larger canopy light transmission correlated with larger windfleck intensity, a larger number of long windflecks, and reduced clustering. As for single‐leaf traits, more erect leaves correlated with higher‐intensity windflecks that occurred more frequently and with shorter intervals between them, as well as reduced clustering. Species with wider leaves tended to experience shorter, lower‐intensity windflecks, while those with longer leaves correlated with a larger light sum during single windflecks.

As plants acclimate to fluctuating light, their leaves tend to become thinner (Morales and Kaiser 2020; Shrestha et al. 2025; Vialet‐Chabrand et al. 2017; Zhang et al. 2020); this may be a strategy to balance the reduction in photosynthetic light use efficiency per unit leaf area by making less “costly” leaves. However, we do not know whether the ability to reduce leaf thickness correlates with relatively stronger growth under sunflecks. Also, it is unclear what difference such reductions in leaf thickness will make in dense crop canopies with high LAI, and where inside crop canopies the strongest changes in leaf thickness occur. The leaf economics spectrum (Wright et al. 2004) may be a useful framework for understanding leaf acclimation to fluctuating light in the future.

To the best of our knowledge, crop growth under light fluctuations resembling sunflecks has not been tested. As stated earlier, studies using faster light intensity fluctuations in plant growth experiments are sparse (Figure 2d). This may be because the effects of fluctuating light on whole‐plant physiology and growth are notoriously hard to grasp. Firstly, quantifying these effects requires a strict *ceteris paribus* approach, wherein plant growth under a fluctuating light regime is compared to that under non‐fluctuating light of the same average intensity, photoperiod, and spectrum (Morales and Kaiser 2020). This generally means experiments in climate chambers, which puts a strong space and time limit on experiments, and is a strong divergence from natural conditions (Poorter et al. 2016). To date, many climate chambers are not equipped with LED lamps that could fluctuate at the relevant frequencies, and even if they are, their control software typically does not allow for such rapid fluctuations. Thus, dedicated setups will have to be built to study plant growth under sunflecks, something that to our knowledge is very rare (although the setup presented by Heuermann et al. (2023) represents an important step). Inspiration for future experiments could be gleaned from research on algae: for example, Graham et al. (2017) grew algae under a large range of frequencies (0.00001–1 Hz) and duty ratios (0.25–0.75), based on which they were able to construct a general model of algae growth under fluctuating light. Although similar experiments are much harder to do in crops (requiring much more space and time), they would be very useful for quantifying the extent to which crop growth is affected by sunflecks.

## 4 Sunflecks and Climate Change: Assessing What We Know

In the face of global climate change, one important aspect of sunfleck photosynthesis is how crops under naturally fluctuating light interact with rising [CO_2_], heat, drought and leaf‐to‐air vapor pressure deficit (VPD). While the effects of these factors on steady‐state *A, g*_s_ and plant growth under constant conditions are well known, the same cannot be said about fluctuating light (Ainsworth and Long 2021). As for the general effects of climate change‐related factors on photosynthesis and crop growth, briefly, rising [CO_2_] reduces the rates of photorespiration in C_3_ crops and thus increases *A*, generally increasing biomass in C_3_ crops (Ainsworth and Long 2021; Poorter et al. 2022). Higher [CO_2_] also reduces *g*_s_, in both C_3_ and C_4_ crops (Ainsworth and Rogers 2007). Heat stress affects myriad biological processes, and broadly reduces biomass through (at least) a reduction in *A*, and increased rates of (photo‐)respiration. Heat can further lead to stomatal uncoupling (i.e., *g*_s_ increases while *A* declines; Wang et al. 2026) which increases the rate of soil moisture loss. Both drought and high VPD often cause stomatal closure and a reduction in *A* and biomass; upon long‐term drought, parts of the photosynthetic machinery, such as Rubisco concentration, may be reduced (Flexas and Medrano 2002). The extent to which plants close their stomata upon drought or high VPD depends on a species' apparent isohydricity (the tendency to maintain leaf water status through strict stomatal and xylem flow regulation; Konings and Gentine 2017), which can be affected by a plants' environment (Feng et al. 2019). Below, we also examine responses to combined changes of VPD and heat, as these two factors are often changed concomitantly during gas exchange experiments.

To assess the effects of these factors on plants under sunflecks, we surveyed the literature: we identified short‐term (minutes–hours; 31 studies; Table S7) and long‐term (days–weeks; 4 studies; Table S8) effects of elevated [CO_2_], heat, drought, VPD, and VPD × heat on dynamic *A* and plant growth under fluctuating light. The relative abundance of studies on short‐term effects allowed a closer look at some processes underlying dynamic *A*, namely the rates of gain and loss of photosynthetic induction, the rates of stomatal opening and closure, as well as post‐illumination CO_2_ gain and burst (Figure S5). We found that rising [CO_2_] predominantly increased the rate of photosynthetic induction (chiefly due to a larger [CO_2_] concentration gradient between the mesophyll and the air surrounding the leaf) and tended to increase post‐illumination CO_2_ gain, while reducing the rate of loss of photosynthetic induction and the post‐illumination CO_2_ burst (due to reduced rates of photorespiration). While it had no clear effects on the rates of stomatal movement, this nevertheless means that rising [CO_2_] benefits the dynamics of *A* under sunflecks (Kaiser et al. 2015; Tomimatsu and Tang 2016). This is further confirmed by plants grown under fluctuating light which, when combined with elevated [CO_2_], showed stronger growth benefits than plants grown under non‐fluctuating light (Leakey et al. 2002; Shrestha et al. 2025; Table S8). Unlike the direct (“substrate”) effect of elevated [CO_2_], acclimation to elevated [CO_2_] did not further alter dynamic responses to fluctuating light (Table S8).

Drought led to faster stomatal closure, but had no clear effects on the rates of photosynthetic induction nor stomatal opening; however, due to faster stomatal closure and reduced *g*_s_ and *g*_m_ in drought‐stressed plants, the reduction of *A* in drought‐stressed plants is likely exacerbated under sunflecks (Zeng et al. 2024). High VPD tended to slow down photosynthetic induction, and increase the rate of stomatal movement. The combination of high VPD and heat, in contrast, had no clear effects on photosynthetic induction nor stomatal opening, and tended to slow down stomatal closure. Finally, heat tended to increase the rate of stomatal closure and the loss of photosynthetic induction and increase the post‐illumination burst. The rates of stomatal opening and photosynthetic induction were, on balance, unaffected by heat.

We tentatively conclude that rising [CO_2_] benefits dynamic *A* and plant growth under sunflecks, while drought, heat, and high VPD likely exacerbate the negative effects of sunflecks on *A* (Figure S5). However, we emphasize that studies using realistic lightflecks are lacking in this area as well, such that our outlook on how climate change affects crop growth under sunflecks may change.

## 5 Conclusions and Outlook

Despite years of work on plant physiology and growth under fluctuating light, we know surprisingly little about the effects of naturally occurring sunflecks on leaf and canopy photosynthesis, and more generally on crop growth in the field. We lack a clear overview of the relative prevalence of wind‐ and sunflecks in crop canopies, which is why we cannot estimate their relative importance for plants in the field. Unlike for single leaves, we do not know how well crop canopies utilize sunflecks, compared to their radiation use efficiency under constant light. We found that several topics are in their infancy, including pulsed light effects on *A*, high‐frequency measurements of irradiance in canopies, and methods for the identification of wind‐ and cloudflecks in irradiance time series; hence, there are many exciting research avenues to be explored in order to better understand *A* and crop growth under fluctuating light. We propose the following research directions.

### 5.1 Better Understanding of Sunflecks and Their Drivers

While we know that wind speed, the direct/diffuse ratio and cloudiness (Nishimura et al. 1998), as well as canopy architecture (Burgess et al. 2021; Durand et al. 2026) affect sunfleck properties, we lack hard data on the relative effects of these factors. Longitudinal studies where light intensities in crop canopies and other weather data are logged at high enough frequencies will be instrumental in determining the role of weather conditions on sunfleck properties. First‐order fluctuations can be quantified following Durand et al. (2021) (Figure 1d–f), however methods for identifying second‐order fluctuations, such as when windflecks are superimposed onto cloudflecks, need to be developed. Properties of wind‐ and cloudflecks should be determined separately to assess their relative importance. Small, lightweight (and possibly wireless) light intensity sensors could be attached to leaves to elucidate whether the uppermost leaves in a canopy experience different light intensity patterns than those below them. Further, synergies with established eddy covariance systems (e.g., FLUXNET; Baldocchi 2020) could be developed, as such systems already collect wind data at high frequencies, as well as estimate canopy photosynthesis at the larger scale, which could be used to ground truth model estimates of canopy photosynthesis under sunflecks. These efforts may result in the construction of mechanistic models that predict sunfleck properties based on environmental factors (and possibly architectural properties); given the broad availability of meteorological data, such models could enable experimenters to estimate the sunfleck exposure in any agricultural field under any weather condition.

### 5.2 Better Elucidation of “Sunfleck Photosynthesis”

As highlighted, very few studies have examined photosynthesis under the frequencies, duty ratios and amplitudes relevant to windflecks; hence, our knowledge of how photosynthesis behaves under windflecks, and which processes limit it, is very limited. Also here, a better separation of the effects of wind‐ and cloudflecks on *A* is needed. By measuring and later modelling photosynthetic CO_2_ uptake and related processes in a range of species (both C_3_ and C_4_), at various frequency × duty ratio × amplitude (× baseline intensity) combinations, and under a range of environmental conditions (most importantly light intensity, [CO_2_], and temperature), we can dramatically increase our knowledge of “windfleck photosynthesis.” Pools of ATP, NADPH and CBB metabolites buffer photosynthesis against light intensity fluctuations, but their importance under windflecks needs to be worked out, especially in C_4_ crops (Stitt and Zhu 2014). We also need to elucidate the role of photorespiration, that is, by modulating the O_2_ concentration, and the role of dynamic photoprotection, under these conditions. Transient limitations during these fluctuations can further be elucidated using a range of tools, such as chlorophyll fluorescence, P^700^ absorbance changes, C^13^ labelling, and photosynthesis mutants. Further, current models of dynamic photosynthesis (listed in Table S4) should be tested using relevant fluctuations as input: can they reproduce *A* under windflecks, and if so, which transient limitations do they predict to limit *A* (if any)? All these efforts should result in new mechanistic insight, culminating in improved models of *A* under sunflecks, and new targets for crop improvement.

### 5.3 Plant Growth and Acclimation Under Fluctuating Light Resembling Windflecks

How plants grow under and acclimate to windflecks is entirely unknown, as even the fastest such fluctuations are much slower than those highlighted here (Table S5). Also, artificial light intensity fluctuations are usually applied at a duty ratio of 0.5, which appears to be much larger than that of windflecks (Figure 3d). Experiments using low‐frequency lightflecks often suggest a growth penalty of fluctuating light, as well as adjustments in leaf thickness (Morales and Kaiser 2020), and it will be exciting to test whether the same phenomena hold under windflecks. Further, to what extent well‐described transformants of Rubisco activase (e.g., *rwt43*; Carmo‐Silva and Salvucci 2013) or stomatal conductance (e.g., EPF2, Franks et al. 2015) affect growth under lightflecks that mimic sunflecks needs to be tested, as this would provide more realistic indications of the effects of speeding up specific processes in the field.

### 5.4 Effects of Concurrent Leaf Temperature and Light Intensity Fluctuations

Due to the energy inherent in light, sudden increases in light intensity also cause leaf temperature (*T*_leaf_) to suddenly rise. These rises can be substantial: in the field, *T*_leaf_ fluctuations of up to 18.3°C have been recorded (Fauset et al. 2018). Since temperature is a major driver of photosynthesis, these rapid *T*_leaf_ fluctuations should additionally affect dynamic *A*. Indeed, moderate changes in *T*_leaf_ that occurred at the same time as step changes in light intensity modulated the rates of photosynthetic induction and stomatal movements (Kang et al. 2020; Shrestha et al. 2026). However, how concurrent *T*_leaf_ and light intensity changes under windflecks affect dynamic *A* is not known.

### 5.5 Implications for Global Photosynthesis

Remote sensing approaches typically estimate canopy‐wide *A* assuming steady‐state (instantaneous) responses to light, and use eddy covariance data to validate these estimates (Ryu et al. 2019). There seems to be good agreement between modelled and measured regional carbon uptake at this scale (Li et al. 2023; Nelson et al. 2024), which deserves critical attention given the supposed reductions in sunfleck *A* relative to steady‐state *A*. Also, sun‐induced fluorescence (SIF) is often used to predict *A*, assuming steady‐state *A* responses to light intensity (Dechant et al. 2020; Porcar‐Castell et al. 2021). How the SIF‐*A* relationship is impacted by fluctuating light is entirely unknown. More work that connects the various measurement and modelling scales, as well as integrates them across disciplines, is needed to tackle these knowledge gaps.

## Author Contributions

**Elias Kaiser:** conceptualization, investigation, writing – original draft, writing – review and editing, methodology, validation, visualization, formal analysis, data curation. **Rui Sun:** investigation, formal analysis, writing – review and editing, validation. **Youngryel Ryu:** conceptualization, writing – review and editing, project administration, supervision, funding acquisition, investigation, resources, formal analysis.

## Funding

This work was supported by the National Research Foundation of Korea, RS‐2024‐00348585 and Rural Development Administration, RS‐2024‐00397146.

## Conflicts of Interest

The authors declare no conflicts of interest.

## Supporting information


**Data S1:** Literature review and data analysis for Figure 2 and Figure S5, Tables S2–S4, S7, and S8.
**Data S2:** Modelling.
**Figure S1:** Percentage of studies mentioning photosynthesis under fluctuating light per year. The search terms *photosynthesis, “fluctuating light”* were entered for per year on Google Scholar (range: 1980–2025). Also, the search term *photosynthesis* was entered for the same period (date of search: 20 February 2026). To obtain a percentage, the number of studies mentioning *photosynthesis, “fluctuating light”* was then divided by the number of studies mentioning *photosynthesis*.
**Figure S2:** Application of sunfleck identification method following Durand et al. (2021) on a sunny (left column), cloudy (middle column) and overcast day (right column). Different rows show various temporal scales.
**Figure S3:** Windfleck amplitude measured in crop canopies, as observed by Durand and co‐workers. For more information, see Table S1.
**Figure S4:** Behavior of the dynamic photosynthesis (*A*) model (Kirschbaum et al. 1998) after parameterization (see Table S5). (A, B) Light response curves of steady‐state *g*_s_ and *A*. (C, D) Induction responses of *g*_s_ and *A*, respectively, following a step increase in PPFD from 50 to 1000 μmol m^−2^ s^−1^ over 20 min. (E, F) Responses of *g*_s_ and *A*, respectively, under repeated cycles of 50 and 1000 μmol m^−2^ s^−1^. Gray shading denotes low‐light periods. For the steady‐state simulations, light‐dependent variables were set directly to their equilibrium values at each light intensity, and the remaining state variables were solved from the steady‐state algebraic system.
**Figure S5:** Overview of short‐term effects (minutes–hours) of environmental factors associated with global climate change on dynamic photosynthesis and its underlying processes, based on a literature overview. Effects on a given process are stated based on an increase in a given environmental factor. Each pie chart represents the number of experimental units reporting either an increase, decrease or no effect of an increase in a given environmental factor on a given process. Processes include the rates of photosynthetic induction and loss of photosynthetic induction, rates of stomatal opening and closure, and time‐integrated post‐illumination CO_2_ gain as well as post‐illumination CO_2_ burst. Analysis is based on 31 research papers reporting 110 experimental units. Experimental units are defined as the response of a given process to an increase in a given environmental factor, per genotype and per research paper (see Table S7 for details).
**Table S1:** Key windfleck properties in crop canopies, as observed by Durand and co‐workers. Windfleck duty ratio was calculated as windfleck frequency * windfleck duration. All studies are listed in Supporting Information References.
**Table S2:** Overview of studies measuring solar light intensity fluctuations within crop canopies. All studies are listed in Supporting Information References.
**Table S3:** Overview of studies using dynamic photosynthesis models to estimate the rate of photosynthesis under fluctuating light intensities. Slowest and fastest fluctuation refer to the minimal and maximal rates of fluctuations used when simulating the dynamics of photosynthesis. All studies are listed in Supporting Information References.
**Table S4:** Overview of studies, and treatments within studies per genotype, of plant growth under fluctuating light. Treatment names are those assigned within a given study, and are listed here to differentiate the different fluctuating light treatments. All studies are listed in Supporting Information References.
**Table S5:** Parameters of the Kirschbaum et al. (1998) photosynthesis model fitted to rice ( *Oryza sativa* L.) data by Yamori et al. (2020); additional parameter sources are indicated in the table. “R fit” denotes parameter estimation from digitized steady‐state response curves in R using the “photosynthesis” package (package version 2.1.5; Stinziano et al. 2024).
**Table S6:** Sensitivity analysis testing the effects of speeding up single processes on time‐integrated dynamic *A*. Components tested were time constants of stomatal opening (*𝜏*_op_) and closure (*𝜏*_cl_), Rubisco activation (*𝜏*_i,R_) and deactivation (*𝜏*_d,R_), as well as of RuBP regeneration activation (*𝜏*_i,fast_) and deactivation (*𝜏*_d,fast_); per analysis, the original value (baseline value) of a given time constant was reduced by 50% (perturbed value).
**Table S7:** Short‐term effects (minutes–hours) of environmental factors associated with global climate change on dynamic photosynthesis and its underlying processes. Processes include the rates of photosynthetic induction and loss of photosynthetic induction, rates of stomatal opening and closure, and time‐integrated post‐illumination CO_2_ gain as well as post‐illumination CO_2_ burst. Effects on a given process are stated based on an increase in a given environmental factor. [CO_2_], CO_2_ concentration; FC, field capacity; kPa, kilopascal; ppm, parts per million; v/v, volume per volume.
**Table S8:** Long‐term effects (days–weeks) of environmental factors associated with global climate change on dynamic photosynthesis and its underlying processes. Processes include plant growth, the rate of photosynthetic induction, rates of stomatal opening and closure, and time‐integrated post‐illumination CO_2_ gain. Effects on a given process are stated based on an increase in a given environmental factor. FL, fluctuating light; ppm, parts per million.

## Data Availability

Data and model code can be found under https://doi.org/10.5061/dryad.f1vhhmhch.
