# Supplementary material for: Overlooked Sunfleck Properties and Potential Implications for Crop Improvement
Source: Glob Chang Biol. 2026 Oct 6;32(10):e71147. doi: 10.1111/gcb.71147 (PMC13731934; doi:10.1111/gcb.71147)
Supplement: Supplementary file 1 [file GCB-32-e71147-s001.docx]

**Supporting information to Kaiser et al.: *Overlooked sunfleck properties and potential implications for crop improvement***

Data S11: Literature review and data analysis for Fig. 2 and Fig. S5, Tables S2-4, S7 and S8

Selection of relevant studies

Google Scholar was searched for different types of studies, using the search terms specified below. Each search was stopped when no relevant paper could be found for three consecutive pages (i.e., no positive hit among 30 studies). Per evaluated study, the title and abstract were screened, relevant papers were downloaded and screened once more using the materials and methods and results sections, using the criteria specified below.

For studies measuring light intensities in crop canopies (Table S2), the search term “fluctuating light, canopy, net radiation” was used on 29 November 2025, generating 35.000 hits, of which the first 250 were evaluated. Furthermore, the search term “fluctuating light, canopy” was used on 30 November 2025, generating 95.000 hits, of which the first 500 were evaluated. Criteria for inclusion were: net radiation was measured in crop canopies, more than once per day. This means that measurements in e.g. forests or algae were excluded. Further, reference lists of included papers were searched for any relevant papers.

For studies using dynamic photosynthesis models (Table S3), the search term “fluctuating light, photosynthesis, model” was used on 30 November 2025, generating 103.000 hits, of which the first 550 were evaluated. Criteria for inclusion were that a model of dynamic leaf or canopy photosynthesis is used to simulate the time course of net CO_2_ exchange at the leaf or whole-canopy level under fluctuations in light intensity. The model works with time constants to describe the dynamic nature of photosynthesis to fluctuations in light intensity.

For studies in which plants were grown under lightflecks (Table S4), the search term “fluctuating light, biomass, plant” was used on 30 November 2025, generating 158.000 hits, of which the first 360 were evaluated. Criteria for inclusion were that plants were grown under fluctuating, reproducible light intensities in a controlled environment.

For studies on the connection between climate change factors and fluctuating light intensity, the search terms “fluctuating light, photosynthesis, *factor*” was used, where “*factor*” was either carbon dioxide, leaf temperature, VPD, or drought. The search was conducted on 4 February 2026. For the factors carbon dioxide (18000 hits), leaf temperature (16800 hits) and VPD (13400), the years 2013 until present were searched, as studies older than 2013 had been included in an earlier review by Kaiser et al. (2015); these were additionally included in the analysis. In the case of drought (49700 hits), all years were included in the search. In each of the four searches, the first 15 Google Scholar pages (150 results) were reviewed.

Data collection and presentation

For studies measuring light intensities in crop canopies, information was collected regarding logging frequency (Hz, no. of logs s^-1^) and logging duration (no. days). Furthermore, information on the type of sunfleck properties that were analyzed in a given study was collected. For studies using dynamic photosynthesis models, information of the slowest as well as fastest light intensity fluctuations used was collected. Furthermore, we noted down whether or not a given study attempted to simulate photosynthesis under field conditions, by which we mean using a large number of different light intensities, amplitudes and frequencies that broadly follow a sinusoidal trajectory, similar to diurnal light intensity changes in the field. For studies in which plants were grown under controlled fluctuations in light intensity, the species, treatment name, number of lightflecks per photoperiod, total light- and shadefleck duration, frequency of fluctuations and their amplitude were noted. Studies whose lightfleck properties could not be estimated unambiguously (e.g. due to complex lightfleck patterns throughout the photoperiod, resulting in large ranges of frequencies, durations and amplitudes) were excluded from this analysis. Key data were presented as boxplots, and outliers were excluded to increase visibility.

For studies on the connection between climate change factors and fluctuating light intensity, the effects of a given environmental factor on plant growth under fluctuating light and/or on several processes underlying dynamic photosynthesis were evaluated per study and per species within a given study. The effect of an increase in an environmental factor on a process was labelled as either “same” (no statistical difference), “increase” (significant increase in the process) or “decrease” (significant reduction in the process).

Date S2: Modelling

We used the dynamic photosynthesis model of Kirschbaum et al. (1998) as the main framework and parameterized it for rice as far as possible using published leaf gas exchange data by Yamori et al. (2020). Because available dynamic g_s_ data were not sufficient to resolve the three interacting stomatal pools in the original Kirschbaum formulation, the stomatal conductance module was replaced with a simpler first-order model following Vico et al. (2011), while the remainder of the model structure was retained.

Actual stomatal conductance approaches its light-dependent equilibrium value at different rates after increases and decreases in irradiance:

$${dg_{s}}/{dt}=\frac{g_{s}^{eq}-g_{s}}{\tau_{s}}, \tau_{s}=\tau_{op} when g_{s}^{eq}>g_{s}; otherwise \tau_{s}=\tau_{cl}$$

The equilibrium light response was retained from the original model. Net photosynthesis rate and stomatal conductance control intercellular CO_2_ concentration (C_i_), which enters the carboxylation calculation below.

Rubisco activity (V_car_) and the fast RuBP-regeneration component (V_f_) approach their light-dependent equilibrium values with process-specific activation and deactivation time constants:

$${dV_{x}}/{dt}=\frac{V_{x}^{eq}-V_{x}}{\tau_{x}} x \in\{V_{car}, V_{f}\}.$$

The model explicitly tracks three metabolite pools. Their rates of change are determined by the production and utilization fluxes of triose phosphates (T), RuBP (R), and glycolate/photorespiratory intermediates (G):

$${dT}/{dt}=V_{e}-V_{T}, {dR}/{dt}=V_{T}-V_{R}, {dG}/{dt}=V_{o}-V_{G}$$

The two fluxes that determine regeneration of the photosynthetic intermediate pools are:

$$V_{e}=V_{j}(1-\frac{T}{T_{max}}), V_{T}=V_{f}\frac{T}{(T+K_{T})} (1-\frac{R}{R_{max}})$$

Intercellular CO_2_ enters the Rubisco-limited carboxylation rate through W_c_; RuBP availability further constrains the actual carboxylation rate:

$$W_{c}=\frac{V_{car}p_{i}^{2}}{(p_{i}+K_{a}) (p_{i}+K_{m})}, V_{c}=W_{c} \frac{R}{(R+K_{R})}$$

Net CO_2_ assimilation is then obtained after accounting for photorespiratory CO_2_ release and non-photorespiratory respiration:

$$A=V_{c}-0.5V_{G}-D_{d}$$

Here, V_e_ and V_T_ are the production and utilization rates of triose phosphates; V_R_, V_o_, and V_G_ are the RuBP-utilization, oxygenation, and glycolate-utilization rates, respectively. Further details of the formulations can be found in Kirschbaum et al. (1998).

Where possible, model parameters were estimated by bounded nonlinear least-squares fitting of simulated steady-state or dynamic responses to the published data. Parameters that could not be estimated directly from the Yamori dataset, or could not be robustly constrained by it, were assigned from supporting literature. Parameter values and sources are listed in Table S5.

After parameterization, several simple simulations were performed, including an *A*/PPFD curve, a g_s_/PPFD curve, a stepwise light intensity change from 50 to 1000 μmol m^-2^ s^-1^ for both *A* and g_s_, and a repeated square-wave lightfleck sequence alternating between 50 and 1000 μmol m^-2^ s^-1^ for 2 min at each level for 10 cycles. These tests showed that the parameterized model reproduced the expected steady-state and dynamic behaviour of *A* and g_s_ with sufficient realism for the subsequent analyses (Fig. S4).

High-frequency light intensity data was recorded in a rice field in Cheorwon, South Korea, during summer 2025. Data were collected using LI-190R sensors (LI-COR, Inc., Lincoln, NE, USA) at 100 Hz. For the present analysis, a representative light sequence recorded on 28 July 2025 was selected to examine how dynamic adjustment and temporal downsampling affected simulated carbon gain under realistically fluctuating light.

Three versions of the same light sequence were prepared: the original 100-Hz full-resolution sequence, and two versions with a 1 minute resolution each. These were a) a downsampled sequence with 1 value logged at the start of a given minute, and b) the mean irradiance over the corresponding 1 minute interval. Furthermore, the light intensity pattern presented by Taylor and Long (2017; their Fig. 6A) was digitized using WebPlotDigitizer (v5.2, Ankit Rohatgi, USA), with manual inspection and correction applied where necessary. Eight simulations were carried out: (1) dynamic model with 100-Hz light pattern, (2) steady-state model with 100-Hz light pattern, (3) dynamic model with the 1-min light pattern, (4) steady-state model with the 1-min light pattern (run twice using either downsampled or mean irradiance), (5) dynamic model with the digitized light pattern (run twice using either downsampled or mean irradiance), and (6) steady-state model with digitized light pattern. In the steady-state simulations, light-dependent processes were set directly to their equilibrium values at each imposed irradiance, and the remaining state variables were solved from steady-state equations.

For each simulation, cumulative *A* was calculated over the selected time window. Dynamic loss was quantified as the difference between cumulative *A* from the steady-state simulation and that from the corresponding dynamic simulation, and relative loss was expressed relative to the steady-state cumulative *A*.

Finally, a sensitivity analysis was carried out for the 100-Hz light sequence. Each time constant was reduced by 50% one at a time, while all other parameters were kept unchanged. The relative loss from each run was then compared with the original value to assess which dynamic components most strongly affected simulated carbon loss.

Unless otherwise stated, all parameter fitting and simulations in this section were carried out in MATLAB (R2024B; MathWorks, Natick, MA, USA).

Supporting information: Figures

Fig. S1. Percentage of studies mentioning photosynthesis under fluctuating light per year. The search terms *photosynthesis, “fluctuating light”* were entered for per year on Google Scholar (range: 1980-2025). Also, the search term *photosynthesis* was entered for the same period (date of search: 20 February 2026). To obtain a percentage, the number of studies mentioning *photosynthesis, “fluctuating light”* was then divided by the number of studies mentioning *photosynthesis*.

Fig. S2. Application of sunfleck identification method following Durand et al. (2021) on a sunny (left column), cloudy (middle column) and overcast day (right column). Different rows show various temporal scales.

Fig. S3. Windfleck amplitude measured in crop canopies, as observed by Durand and co-workers. For more information, see Table S1.

Fig. S4. Behaviour of the dynamic photosynthesis (*A*) model (Kirschbaum et al., 1997) after parameterization (see Table S5). A, B) Light response curves of steady-state g_s_ and *A*. C, D) Induction responses of g_s_ and *A*, respectively, following a step increase in PPFD from 50 to 1000 μmol m^-2^ s^-1^ over 20 min. E, F) Responses of g_s_ and *A*, respectively, under repeated cycles of 50 and 1000 μmol m^-2^ s^-1^. Grey shading denotes low-light periods. For the steady-state simulations, light-dependent variables were set directly to their equilibrium values at each light intensity, and the remaining state variables were solved from the steady-state algebraic system.

Fig. S5. Overview of short-term effects (minutes-hours) of environmental factors associated with global climate change on dynamic photosynthesis and its underlying processes, based on a literature overview. Effects on a given process are stated based on an increase in a given environmental factor. Each pie chart represents the number of experimental units reporting either an increase, decrease or no effect of an increase in a given environmental factor on a given process. Processes include the rates of photosynthetic induction and loss of photosynthetic induction, rates of stomatal opening and closure, and time-integrated post-illumination CO_2_ gain as well as post-illumination CO_2_ burst. Analysis is based on 31 research papers reporting 110 experimental units. Experimental units are defined as the response of a given process to an increase in a given environmental factor, per genotype and per research paper (see Table S7 for details).

Supporting information: Tables

Table S1. Key windfleck properties in crop canopies, as observed by Durand and co-workers. Windfleck duty ratio was calculated as windfleck frequency * windfleck duration. All studies are listed in supplementary references.

| Study | Genotype | Frequency (Hz) | Windfleck duration (s) | Windfleck amplitude (μmol m^-2^ s^-1^) | Duty ratio |
| --- | --- | --- | --- | --- | --- |
| Durand *et al*., 2021 | *Avena sativa* | 2.30 | 0.09 | 8.8 | 0.22 |
|  | *Hordeum vulgare* | 2.50 | 0.09 | 42.2 | 0.21 |
|  | *Vicia faba* | 3.50 | 0.10 | 91.5 | 0.35 |
| Durand *et al*., 2023 | *Hordeum vulgare* | 1.61 | 0.23 | 379.8 | 0.37 |
|  | *Vicia faba* | 0.77 | 0.21 | 163.3 | 0.16 |
|  | *Avena sativa* | 0.92 | 0.27 | 229.8 | 0.24 |
|  | *Brassica napus* | 2.23 | 0.18 | 255.8 | 0.39 |
| Durand *et al*., 2026 | *Triticum aestiva* cv. Crusoe | 1.23 | 0.18 | 98.1 | 0.22 |
|  | *Triticum aestiva* cv. KWS Extase | 1.30 | 0.19 | 154.1 | 0.25 |
|  | *Triticum aestiva* cv. KWS Firefly | 1.46 | 0.23 | 202.8 | 0.34 |
|  | *Triticum aestiva* cv. KWS Kinetic | 0.85 | 0.22 | 108.2 | 0.19 |
|  | *Triticum aestiva* cv. Mayflower | 1.77 | 0.17 | 108.3 | 0.30 |
|  | *Triticum aestiva* cv. RGT Wilkinson | 1.15 | 0.16 | 89.4 | 0.18 |
|  | *Triticum aestiva* cv. Skyfall | 2.18 | 0.22 | 281.4 | 0.48 |
|  | *Triticum aestiva* cv. SY Insitor | 1.45 | 0.17 | 221.5 | 0.25 |
|  | *Triticum aestiva* cv. Theodore | 1.58 | 0.20 | 139.4 | 0.31 |
|  | *Triticum aestiva* cv. Zoom | 1.35 | 0.16 | 311.9 | 0.22 |

Table S2. Overview of studies measuring solar light intensity fluctuations within crop canopies. All studies are listed in supplementary references.

| **Study** | **Species** | **Logging frequency (Hz)** | **Logging duration (days)** | **Sunfleck characteristics analysed** | **Notes** |
| --- | --- | --- | --- | --- | --- |
| Adachi et al., 2019 | Rice | 0.10 | 1.00 | None |  |
| Barradas and Jones, 1996 | Bean | 0.03 | 0.08 | None |  |
| Barradas et al., 1998 | Bean | 0.20 | 53.00 | SF number, SF duration, time fraction |  |
| Burgess et al., 2021 | Wheat | 3.00 | 3.00 | SF duration, SF amplitude, time between SFs | Measurements made within 3 hours of solar noon |
| Dokoozlian and Kliewer, 1995 | Grapevine | 0.02 | 35.00 | Percent canopy area receiving SFs | Measurements made on clear days, within 1 hour of solar noon; logging duration was guessed from vague description; all PAR >700 umol m^-2^ s^-1^ was considered sunfleck |
| Durand et al., 2021 | Oat, barley, broad bean | 100.00 | 0.00 | SF duration, SF amplitude, time between SFs, integrated PAR during SF | Measurement duration per crop was 100 s; Data were recorded between 10:30 and 15:30, in the absence of clouds |
| Durand and Robson, 2023 | Oat, rapeseed, barley, broad bean | 100.00 | 0.00 | SF duration, SF amplitude, time between SFs, integrated PAR during SF, clustering | Measurement duration per crop was 100 seconds; measurements were done close to solar noon, in the absence of clouds |
| Durand et al., 2026 | Winter wheat | 100.00 | 0.00 | SF duration, SF amplitude, time between SFs, integrated PAR during SF, clustering | Measurement duration per winter wheat cultivar was 100 seconds; measurements were done close to solar noon, in the absence of clouds |
| Gutschick et al.,1985 | Sorghum | NA | 2.00 | None | Measurement frequency not clearly described, possibly 1 Hz |
| Impens et al., 1970 | Oat, bean, sunflower, maize | 0.01 | 0.04 | None | Measurements for 1 h, data logged every 100 s |
| Kaiser et al., 2018 | Wheat, mustard | 1.00 | 0.25 | SF duration |  |
| Lakso and Barnes, 1978 | Apple | NA | 0.00 | None | Measurements of 75 s duration are shown, logging frequency not clearly described |
| Li et al., 2025 | Cotton | 0.20 | 1.00 | SF number, SF amplitude |  |
| Nishimura et al., 1998 | Rice | 50.00 | 4.00 | Power spectra of PPFD | Measurements of a representative sunny and overcast day are shown |
| Pearcy et al., 1990 | Soybean | 20.00 | 11.50 | SF number, SF percentage of total PPFD, integrated PPFD during sunflecks, SF duration, SF clusters, duration between SF, SF amplitude | Measurements only on cloudless days, at 1 and 20 Hz |
| Peressotti et al., 2001 | Wheat, sunflower, maize | 8.00 | 19.00 | SF frequency, SF duration, coefficient of variability of PPFD | Measurements done for 16 s at each starting hour during the day |
| Sikma et al., 2020 | Rice | 0.02 | 6.00 | None |  |
| Tang et al., 1988 | *Miscanthus sinensis* | 0.20 | 3.00 | SF duration, SF amplitude |  |
| Tatsumi et al., 2020 | Rice | 0.02 | 16.00 | None |  |
| Tong and Hipps, 1996 | Alfalfa | 27.00 | 21.00 | Power spectra of PPFD | Different sensors with somewhat different specs were used, in two Alfalfa fields; range of frequencies was 10-27 Hz |
| Zheng et al., 2023 | Maize | NA | 1.00 | SF amplitude, SF duration, time between SF |  |

Table S3. Overview of studies using dynamic photosynthesis models to estimate the rate of photosynthesis under fluctuating light intensities. Slowest and fastest fluctuation refer to the minimal and maximal rates of fluctuations used when simulating the dynamics of photosynthesis. All studies are listed in supplementary references.

| **Study** | **Slowest lightfleck (Hz)** | **Fastest lightfleck (Hz)** | **Phot. under field conditions simulated?*** | **Notes** |
| --- | --- | --- | --- | --- |
| Bellasio, 2019 | 0.00 | 0.00 | No |  |
| Gross, 1982 | 0.03 | 0.40 | No |  |
| Gross et al., 1991 | 0.00 | 0.07 | No |  |
| Jishi et al., 2015 | 0.10 | 1000.00 | No |  |
| Joubert et al., 2023 | 0.02 | NA | No |  |
| Kirschbaum et al., 1998 | 0.00 | 0.20 | No |  |
| Laisk et al., 2006 | 0.01 | 0.02 | No |  |
| Morales et al., 2018 | 0.00 | 20.00 | No |  |
| Mott and Woodrow, 2000 | 0.00 | 0.00 | No |  |
| Naumburg et al., 2001 | 0.20 | 0.20 | Yes |  |
| Naumburg and Ellsworth, 2002 | 0.20 | 0.20 | Yes |  |
| Noe and Giersch, 2004 | NA | NA | Yes |  |
| Ooba et al., 2003 | 0.00 | 1.00 | No |  |
| Ozturk et al., 2003 | 0.00 | NA | No |  |
| Pearcy et al., 1997 | 0.00 | 1.00 | No |  |
| Peri et al., 2006 | 0.00 | 0.00 | No |  |
| Porcar-Castell et al., 2006 | 0.01 | 10.00 | No | Model estimates ETR, not photosynthesis |
| Roden, 2003 | 0.00 | 10.00 | No |  |
| Salter et al., 2019 | 0.00 | 0.03 | No |  |
| Salvatori et al., 2022 | 0.00 | 0.03 | No |  |
| Shao et al., 2024 | 0.01 | 0.01 | No |  |
| Stegemann et al., 1999 | 0.00 | 0.67 | Yes | *Fluctuations recreated using artificial light source, changes every 1.5 s |
| Taylor and Long, 2017 | 0.02 | 0.02 | No |  |
| Timm et al., 2004 | 0.02 | 0.02 | No |  |
| Vialet-Chabrand et al., 2016 | 0.00 | NA | No | Photosynthesis under FL is calculated and measured, but it is not clear how often light intensity changed |
| Wang et al., 2020 | 0.10 | 0.10 | Yes |  |
| Wang et al., 2021 | 0.00 | 0.00 | No |  |
| Wang and Zhu, 2025 |  | 0.00 | No |  |
| Zhu et al., 2013 | 0.00 | 0.01 | No |  |

*including various light intensities x frequencies, as can be found within canopies in the field

Table S4. Overview of studies, and treatments within studies per genotype, of plant growth under fluctuating light. Treatment names are those assigned within a given study, and are listed here to differentiate the different fluctuating light treatments. All studies are listed in supplementary references.

| Study | Species | Treatment name | No. lightflecks photoperiod^-1^ | Total lightfleck duration (h) | Total shadefleck duration (h) | Lightfleck frequency (Hz) | Lightfleck amplitude (μmol m^-2^ s^-1^) | Notes |
| --- | --- | --- | --- | --- | --- | --- | --- | --- |
| Alter et al., 2012 | *Arabidopsis thaliana* | SSF650/6 | 120.0 | 0.67 | 11.33 | 0.0028 | 600 |  |
| Alter et al., 2012 | *Arabidopsis thaliana* | SSF1250/12 | 60.0 | 0.33 | 11.67 | 0.0014 | 1200 |  |
| Alter et al., 2012 | *Arabidopsis thaliana* | SSF1250/6 | 120.0 | 0.67 | 11.33 | 0.0028 | 1200 |  |
| Bhuyian & van Iersel, 2021 | Lettuce cv “Little Gem” | 0/400 | 32.0 | 8.00 | 8.00 | 0.0006 | 400 |  |
| Bhuyian & van Iersel, 2021 | Lettuce cv “Little Gem” | 40/360 | 32.0 | 8.00 | 8.00 | 0.0006 | 320 |  |
| Bhuyian & van Iersel, 2021 | Lettuce cv “Little Gem” | 80/320 | 32.0 | 8.00 | 8.00 | 0.0006 | 240 |  |
| Bhuyian & van Iersel, 2021 | Lettuce cv “Little Gem” | 120/280 | 32.0 | 8.00 | 8.00 | 0.0006 | 160 |  |
| Bhuyian & van Iersel, 2021 | Lettuce cv “Little Gem” | 160/240 | 32.0 | 8.00 | 8.00 | 0.0006 | 80 |  |
| Bhuyian & van Iersel, 2021 | Lettuce cv “Green salad bowl” | 0/400 | 32.0 | 8.00 | 8.00 | 0.0006 | 400 |  |
| Bhuyian & van Iersel, 2021 | Lettuce cv “Green salad bowl” | 40/360 | 32.0 | 8.00 | 8.00 | 0.0006 | 320 |  |
| Bhuyian & van Iersel, 2021 | Lettuce cv “Green salad bowl” | 80/320 | 32.0 | 8.00 | 8.00 | 0.0006 | 240 |  |
| Bhuyian & van Iersel, 2021 | Lettuce cv “Green salad bowl” | 120/280 | 32.0 | 8.00 | 8.00 | 0.0006 | 160 |  |
| Bhuyian & van Iersel, 2021 | Lettuce cv “Green salad bowl” | 160/240 | 32.0 | 8.00 | 8.00 | 0.0006 | 80 |  |
| Blom and Zheng, 2009 | Chrysanthemum | 2 mm s^-1^ | 25.9 | 1.80 | 16.20 | 0.0004 | 193 | a |
| Blom and Zheng, 2009 | Chrysanthemum | 8 mm s^-1^ | 103.7 | 1.80 | 16.20 | 0.0016 | 193 | a |
| Blom and Zheng, 2009 | Chrysanthemum | 20 mm s^-1^ | 259.2 | 1.80 | 16.20 | 0.0040 | 193 | a |
| Blom and Zheng, 2009 | Petunia | 2 mm s^-1^ | 25.9 | 1.80 | 16.20 | 0.0004 | 193 | a |
| Blom and Zheng, 2009 | Petunia | 8 mm s^-1^ | 103.7 | 1.80 | 16.20 | 0.0016 | 193 | a |
| Blom and Zheng, 2009 | Petunia | 20 mm s^-1^ | 259.2 | 1.80 | 16.20 | 0.0040 | 193 | a |
| Blom and Zheng, 2009 | Rose | 2 mm s^-1^ | 25.9 | 1.80 | 16.20 | 0.0004 | 193 | a |
| Blom and Zheng, 2009 | Rose | 8 mm s^-1^ | 103.7 | 1.80 | 16.20 | 0.0016 | 193 | a |
| Blom and Zheng, 2009 | Rose | 20 mm s^-1^ | 259.2 | 1.80 | 16.20 | 0.0040 | 193 | a |
| Blom and Zheng, 2009 | Tomato | 2 mm s^-1^ | 25.9 | 1.80 | 16.20 | 0.0004 | 193 | a |
| Blom and Zheng, 2009 | Tomato | 8 mm s^-1^ | 103.7 | 1.80 | 16.20 | 0.0016 | 193 | a |
| Blom and Zheng, 2009 | Tomato | 20 mm s^-1^ | 259.2 | 1.80 | 16.20 | 0.0040 | 193 | a |
| Burgess et al., 2023 | *Arabidopsis thaliana* Ler | FL | 13.1 | 7.66 | 4.34 | 0.0003 | 230 | c |
| Burgess et al., 2023 | *Arabidopsis thaliana* Ws | FL | 13.1 | 7.66 | 4.34 | 0.0003 | 230 | c |
| Dieleman et al., 2025 | Tomato | hour 200/0 | 2.0 | 8.00 | 8.00 | 0.0000 | 200 |  |
| Dieleman et al., 2025 | Tomato | 30 min 125/75 | 16.0 | 8.00 | 8.00 | 0.0003 | 50 |  |
| Dieleman et al., 2025 | Tomato | 30 min 175/25 | 16.0 | 8.00 | 8.00 | 0.0003 | 150 |  |
| Dieleman et al., 2025 | Tomato | 30 min 200/0 | 16.0 | 8.00 | 8.00 | 0.0003 | 200 |  |
| Dieleman et al., 2025 | Tomato | 1 min 125/75 | 480.0 | 8.00 | 8.00 | 0.0083 | 50 |  |
| Dieleman et al., 2025 | Tomato | 1 min 175/25 | 480.0 | 8.00 | 8.00 | 0.0083 | 150 |  |
| Dieleman et al., 2025 | Tomato | 1 min 200/0 | 480.0 | 8.00 | 8.00 | 0.0083 | 200 |  |
| Ding et al., 2019 | Tomato | M | 3.9 | 6.61 | 5.39 | 0.0001 | 85 | c |
| Ding et al., 2019 | Tomato | A | 4.0 | 6.21 | 5.79 | 0.0001 | 85 | c |
| Ding et al., 2019 | Tomato | D | 3.6 | 8.79 | 3.21 | 0.0001 | 85 | c |
| Durand et al., 2024 | *Arabidopsis thaliana* | FHn | 576.0 | 8.00 | 8.00 | 0.0100 | 480 |  |
| Durand et al., 2024 | *Arabidopsis thaliana* | FHw | 576.0 | 8.00 | 8.00 | 0.0100 | 680 |  |
| Durand et al., 2024 | *Arabidopsis thaliana* | FL | 576.0 | 8.00 | 8.00 | 0.0100 | 400 |  |
| Fan et al., 2025 | Soybean | ND12 | 5.6 | 4.50 | 7.50 | 0.0001 | 1050 |  |
| Fan et al., 2025 | Soybean | GX 7 | 5.6 | 4.50 | 7.50 | 0.0001 | 1050 |  |
| Garcia-Molina and Leister, 2020 | *Arabidopsis thaliana* | FL | 120.0 | 2.00 | 10.00 | 0.0028 | 450 |  |
| Heuermann et al., 2024 | *Arabidopsis thaliana* | Exp 1 | 170.0 | 0.04 | 0.05 | 0.0030 | 300 |  |
| Heuermann et al., 2024 | *Arabidopsis thaliana* | Exp 2 | 170.0 | 0.08 | 0.11 | 0.0030 | 300 |  |
| Hubbart et al., 2018 | Rice | FL | 120.0 | 6.00 | 6.00 | 0.0028 | 1400 | b |
| Jezek et al., 2021 | *Arabidopsis thaliana* | FL | 4.5 | 4.50 | 4.50 | 0.0001 | 125 |  |
| Kaiser et al., 2018 | Tomato | FL | 192.0 | 1.07 | 14.93 | 0.0033 | 414 |  |
| Kaiser et al., 2020 | *Arabidopsis thaliana* | FL | 144.0 | 2.40 | 9.60 | 0.0033 | 810 |  |
| Kaiser et al., 2024 | Basil | Square | 8.0 | 8.00 | 8.00 | 0.0001 | 350 |  |
| Kaiser et al., 2024 | Basil | EP | 8.0 | 8.00 | 8.00 | 0.0001 | 125 |  |
| Kaiser et al., 2024 | Pakchoi | Square | 8.0 | 8.00 | 8.00 | 0.0001 | 350 |  |
| Kaiser et al., 2024 | Pakchoi | EP | 8.0 | 8.00 | 8.00 | 0.0001 | 125 |  |
| Kaiser et al., 2024 | Rucola | Square | 8.0 | 8.00 | 8.00 | 0.0001 | 350 |  |
| Kaiser et al., 2024 | Rucola | EP | 8.0 | 8.00 | 8.00 | 0.0001 | 125 |  |
| Kaiser et al., 2024 | Spinach | Square | 8.0 | 8.00 | 8.00 | 0.0001 | 350 |  |
| Kaiser et al., 2024 | Spinach | EP | 8.0 | 8.00 | 8.00 | 0.0001 | 125 |  |
| Kimura et al., 2020 | *Arabidopsis thaliana* | 3 switches | 1.0 | 4.00 | 8.00 | 0.0000 | 440 |  |
| Kimura et al., 2020 | *Arabidopsis thaliana* | 48 switches | 48.0 | 4.00 | 8.00 | 0.0011 | 440 |  |
| Kubasek et al., 2013 | T. aestivum | FL | 154.8 | 5.25 | 10.75 | 0.0027 | 855 |  |
| Kubasek et al., 2013 | S. macrostachya | FL | 154.8 | NA | NA | 0.0027 | 855 |  |
| Kubasek et al., 2013 | C. argentea | FL | 154.8 | NA | NA | 0.0027 | 855 |  |
| Kubasek et al., 2013 | A. caudatus | FL | 154.8 | NA | NA | 0.0027 | 855 |  |
| Leakey et al., 2002 | *Shorea leprosula* | FL | 72.6 | 3.63 | 8.97 | 0.0016 | 495 |  |
| Lei et al., 2025 | Cotton | LFL | 6.0 | 6.00 | 6.00 | 0.0001 | 100 |  |
| Lei et al., 2025 | Cotton | HFL | 45.0 | 6.00 | 6.00 | 0.0010 | 100 |  |
| Qiao et al., 2021 | Maize | 1CF | 1.0 | 5.00 | 5.00 | 0.0000 | 1550 |  |
| Qiao et al., 2021 | Maize | 10CF | 10.0 | 5.00 | 5.00 | 0.0003 | 1550 |  |
| Qiao et al., 2021 | Maize | 100CF | 100.0 | 5.00 | 5.00 | 0.0028 | 1550 |  |
| Sakoda et al., 2020 | *Arabidopsis thaliana* | 3 switches | 1.0 | 4.00 | 8.00 | 0.0000 | 440 |  |
| Sakoda et al., 2020 | *Arabidopsis thaliana* | 48 switches | 48.0 | 4.00 | 8.00 | 0.0011 | 440 |  |
| Shao et al., 2020 | Lettuce | A3 | 3.0 | 1.00 | 15.00 | 0.0001 | 350 |  |
| Shao et al., 2020 | Lettuce | A6 | 6.0 | 1.00 | 15.00 | 0.0001 | 350 |  |
| Shao et al., 2020 | Lettuce | A12 | 12.0 | 1.00 | 15.00 | 0.0002 | 350 |  |
| Shimadzu et al., 2019 | *Arabidopsis thaliana* | 3 switches | 1.0 | 4.00 | 8.00 | 0.0000 | 440 |  |
| Shimadzu et al., 2019 | *Arabidopsis thaliana* | 48 switches | 48.0 | 4.00 | 8.00 | 0.0011 | 440 |  |
| Shrestha et al., 2025 | Cucumber | FL | 144.0 | 8.00 | 8.00 | 0.0025 | 260 | c |
| Thormaelen et al., 2017 | *Arabidopsis thaliana* | FL | 192.0 | 3.20 | 12.80 | 0.0033 | 810 |  |
| Watling et al., 1997 | *Alocasia macrorrhiza* | FL | 10.0 | 1.67 | 10.33 | 0.0002 | 450 |  |
| Watling et al., 1997 | D. diphyllostegia | FL | 10.0 | 1.67 | 10.33 | 0.0002 | 450 |  |
| Watling et al., 1997 | M. minutum | FL | 10.0 | 1.67 | 10.33 | 0.0002 | 450 |  |
| Watling et al., 1997 | O. novo-guinensis | FL | 10.0 | 1.67 | 10.33 | 0.0002 | 450 |  |
| Woodford et al., 2025 | *Setaria viridis* | FL | 160.0 | 2.67 | 13.33 | 0.0028 | 750 |  |
| Wu et al., 2023 | Maize | FL300 | 1.0 | 5.00 | 5.00 | 0.0000 | 1550 |  |
| Wu et al., 2023 | Maize | FL30 | 10.0 | 5.00 | 5.00 | 0.0003 | 1550 |  |
| Wu et al., 2023 | Maize | FL3 | 100.0 | 5.00 | 5.00 | 0.0028 | 1550 |  |
| Yamori et al., 2020 | Rice | C | 1.0 | 8.00 | 8.00 | 0.0000 | 650 |  |
| Yamori et al., 2020 | Rice | FL | 48.0 | 8.00 | 8.00 | 0.0008 | 650 |  |
| Zhang et al., 2020 | Tomato | FL | 96.0 | 8.00 | 8.00 | 0.0017 | NA |  |
| Zheng et al., 2006 | Gerbera | FL | 581.5 | 2.31 | 15.69 | 0.0090 | NA |  |
|  |  |  |  |  |  |  |  |  |
| ^a^Fluctuation durations estimated based on assumed lamp illumination area | | | | | | |  |  |
| ^b^Ramping up and down of PAR at edges of photoperiod ignored in calculations | | | | | | |  |  |
| ^c^All fluctuation characteristics averaged | | | |  |  |  |  |  |

Table S5. Parameters of the Kirschbaum et al. (1997) photosynthesis model fitted to rice (*Oryza sativa* L.) data by Yamori et al. (2020); additional parameter sources are indicated in the table. “R fit” denotes parameter estimation from digitized steady-state response curves in R using the “photosynthesis” package (package version 2.1.5; Stinziano et al., 2024).

| **Symbol** | **Description** | **Value** | **Unit** | **Source / estimation** |
| --- | --- | --- | --- | --- |
| Stomatal conductance model | | | | |
| g_max_ | maximum stomatal conductance | 0.531 | mol_H2O_ m^-2^ s^-1^ | based on Yamori et al., 2020 Fig. 1c, value at 1500 µmol m^-2^ s^-1^ |
| g_min_ | minimum stomatal conductance | 0.036 | mol_H2O_ m^-2^ s^-1^ | based on Yamori et al., 2020 Fig. 1c, value at 0 µmol m^-2^ s^-1^ |
| α_gs_ | initial slope of stomatal conductance-PAR relationship | 0.001 | mol_H2O_ µmol_photons_^-1^ | slope between 0 and 50 µmol m^-2^ s^-1^ from Yamori et al. 2020 Fig. 1c |
| θ_gs_ | curvature of stomatal conductance-PAR relationship | 0.885 | - | estimated by fitting a non-rectangular hyperbola to the digitized gs–PAR curve from Yamori Fig. 1c |
| τ_op_ | effective stomatal opening time constant | 189 | s | digitized g_s_ time series from Yamori Fig. 4b; fitted using a first-order stomatal model (Vico et al., 2011) |
| τ_cl_ | effective stomatal closing time constant | 225 | s | same as τ_op_ |
| Photosynthesis model | | | | |
| V_cmax_ | maximum Rubisco activity in high light | 100.3 | µmol_CO2_ m^-2^ s^-1^ | Yamori et al., 2020 Fig. 1b; R fit |
| V_cmin_ | minimum Rubisco activity in high light | 10 | µmol_CO2_ m^-2^ s^-1^ | 0.1V_cmax_ (Naumburg et al., 2001) |
| α_R_ | initial slope of Rubisco activity-PAR relationship | 0.13 | mol_CO2_ mol_photons_^-1^ | Naumburg et al., 2001 |
| θ_R_ | curvature of Rubisco activity-PAR relationship | 0.9 | - | Gross et al., 1991 |
| τ_i,R_ | time constant for Rubisco activation | 161 | s | digitized Rubisco activation time series from Yamori et al., 2020 Fig. S4C; incomplete deactivation was handled by fixing the lower asymptote to the observed minimum (equal to the starting value). |
| τ_d,R_ | time constant for Rubisco deactivation | 450 | s | same as τ_i,R_ |
| V_jmax_ | maximum electron transport rate | 230 | µmol_electrons_ m^-2^ s^-1^ | Yamori et al. 2020 Fig. 1b; R fit |
| α_Vj_ | initial slope of electron transport-PAR relation | 0.07 | mol_electrons_ mol_photons_^-1^ | Yamori et al. 2020 Fig. 1d; R fit |
| θ_Vj_ | curvature of electron transport-PAR relationship | 0.85 | - | default; R fit |
| F_max_ | maximum RuBP regeneration rate | 180.5 | µmol_RuBP_ m^-2^ s^-1^ | 1.8V_cmax_ (Naumburg et al., 2001) |
| F_min_ | minimum RuBP regeneration rate | 0.4 | µmol_RuBP_ m^-2^ s^-1^ | 0.2% of V_fmax_ (Naumburg et al., 2001) |
| α_Vf_ | initial slope of RuBP regeneration-PAR relationship | 0.3 | mol_RuBP_ mol_photons_^-1^ | Naumburg et al., 2001 |
| θ_Vf_ | curvature of RuBP regeneration-PAR relationship | 0.95 | - | Naumburg et al., 2001 |
| τ_i,fast_ | time constant for RuBP regeneration activation | 90 | s | Sassenrath-Cole & Pearcy, 1994 |
| τ_d,fast_ | time constant for RuBP regeneration deactivation | 180 | s | Sassenrath-Cole & Pearcy, 1994 |
| R_max_ | maximum RuBP pool size | 100.3 | µmol_RuBP_ m^-2^ | 1×Vcmax (Naumburg et al., 2001) |
| T_max_ | maximum triose phosphate pool size | 50 | µmol_triosephosphate_ m^-2^ | ~0.5×Vcmax (Naumburg et al., 2001) |
| K_R_ | Michaelis-Menten constant for utilisation of RuBP by Rubisco | 27.5 | µmol_RuBP_ m^-2^ | Pearcy et al., 1997 |
| K_T_ | Michaelis-Menten constant for utilisation of RuBP for triose phosphate | 20 | µmol_triosephosphate_ m^-2^ | Pearcy et al., 1997 |
| R_d_ | non-photorespiratory day respiration | 1.4 | µmol_CO2_ m^-2^ s^-1^ | Yamori et al. 2020 Fig. 1d; R fit |
| τ_g_ | time constant for glycolate pool decay | 20 | s | Naumburg et al., 2001 |
| Γ* | CO_2_ compensation point in absence of R_d_ | 43.6 | µmol_CO2_ mol_air_^-1^ | Hermida-Carrera et al., 2016 |
| K_a_ | Michaelis-Menten constant for Rubisco activation by CO_2_ | 10 | μbar | Kirschbaum & Farquhar, 1984 |
| K_c_ | Michaelis-Menten constant of Rubisco for carboxylation | 239 | μbar | Makino et al., 1988 |
| K_o_ | Michaelis-Menten constant of Rubisco for oxygenation | 266 | mbar | Makino et al., 1988 |

Table S6. Sensitivity analysis testing the effects of speeding up single processes on time-integrated dynamic *A*. Components tested were time constants of stomatal opening (𝜏_op_) and closure (𝜏_cl_), Rubisco activation (𝜏_i, R_) and deactivation (𝜏_d, R_), as well as of RuBP regeneration activation (𝜏_i, fast_) and deactivation (𝜏_d, fast_); per analysis, the original value (baseline value) of a given time constant was reduced by 50% (perturbed value).

| **Parameter** | **Baseline value (s)** | **Perturbed value (s)** | **Relative loss (%)** | **Δ relative loss (%)** | **Change vs. baseline loss (%)** |
| --- | --- | --- | --- | --- | --- |
| 𝜏_op_ | 189 | 94.5 | 0.62 | -0.23 | -27.74 |
| 𝜏_cl_ | 225 | 112.5 | 1.0 | 0.16 | 18.84 |
| 𝜏_i, R_ | 161 | 80.5 | -0.93 | -1.78 | -208.80 |
| 𝜏_d, R_ | 450 | 225 | 2.23 | 1.38 | 162.07 |
| 𝜏_i, fast_ | 90 | 45 | 0.45 | -0.40 | -47.38 |
| 𝜏_d, fast_ | 180 | 90 | 1.41 | 0.56 | 65.14 |

Note: The baseline relative loss over the entire day was 0.85% (Fig. 4B). For each sensitivity run, only one time constant was reduced by 50%, while all other parameters were kept at their baseline values. Change in loss (%) was calculated relative to the baseline loss. Negative values indicate lower loss than the baseline simulation, and therefore improved simulated photosynthetic performance.

Table S7. Short-term effects (minutes-hours) of environmental factors associated with global climate change on dynamic photosynthesis and its underlying processes. Processes include the rates of photosynthetic induction and loss of photosynthetic induction, rates of stomatal opening and closure, and time-integrated post-illumination CO_2_ gain as well as post-illumination CO_2_ burst. Effects on a given process are stated based on an increase in a given environmental factor. Abbreviations: [CO_2_], CO_2_ concentration; FC, field capacity; kPa, kilopascal; ppm, parts per million; v/v, volume per volume.

| **Study** | **Environmental factor** | **Process** | **Species** | **Factor levels** | **Effect on process** |
| --- | --- | --- | --- | --- | --- |
| Ariura et al., 2023 | [CO_2_] | Photosynthetic induction | *Fagus crenata* | ambient, 700 ppm | faster |
| Ariura et al., 2023 | [CO_2_] | Stomatal opening | *Fagus crenata* | ambient, 700 ppm | faster |
| Caine et al., 2025 | [CO_2_] | Stomatal closure | Wheat | 450, 720 ppm | same |
| Caine et al., 2025 | [CO_2_] | Stomatal opening | Wheat | 450, 720 ppm | slower |
| Caine et al., 2025 | [CO_2_] | Photosynthetic induction | Wheat | 450, 720 ppm | same |
| Caine et al., 2025 | VPD x heat | Stomatal closure | Wheat | 1.43 kPA x 27 °C, 3.14 kPA @ 37 °C | slower |
| Caine et al., 2025 | VPD x heat | Stomatal opening | Wheat | 1.43 kPA x 27 °C, 3.14 kPA @ 37 °C | slower |
| Caine et al., 2025 | VPD x heat | Photosynthetic induction | Wheat | 1.43 kPA x 27 °C, 3.14 kPA @ 37 °C | faster |
| Carmo-Silva and Salvucchi, 2013 | Heat | Photosynthetic induction | *Arabidopsis thaliana* | 23, 35 °C | slower |
| Chazdon and Pearcy, 1986 | [CO_2_] | Photosynthetic induction | *Alocasia macrorrhiza* | 200, 640, 844 ppm | faster |
| Clemente and Marler, 1996 | Drought | Stomatal closure | Papaya | 100%, 57% FC | faster |
| Clemente and Marler, 1996 | Drought | Stomatal opening | Papaya | 100%, 57% FC | same |
| Clemente and Marler, 1996 | Drought | Photosynthetic induction | Papaya | 100%, 57% FC | slower |
| Doehlert et al., 1979 | [CO_2_] | Post-illumination CO_2_ burst | Wheat | 0-10 uM | decrease |
| Doehlert et al., 1979 | Heat | Post-illumination CO_2_ burst | Wheat | 25-40 °C | increase |
| Huang et al., 2017 | Heat | Photosynthetic induction | Rice | 28, 40 °C | slower |
| Huang et al., 2017 | Heat | Stomatal opening | Rice | 28, 40 °C | same |
| Kaiser et al., 2017a | [CO_2_] | Photosynthetic induction | Tomato | 400, 800 ppm | faster |
| Kaiser et al., 2017a | [CO_2_] | Stomatal opening | Tomato | 400, 800 ppm | same |
| Kaiser et al., 2017a | Heat | Photosynthetic induction | Tomato | 22.8, 30.5 °C | same |
| Kaiser et al., 2017a | Heat | Stomatal opening | Tomato | 22.8, 30.5 °C | same |
| Kaiser et al., 2017a | VPD | Photosynthetic induction | Tomato | 0.8, 1.5, 2.3 kPa | slower |
| Kaiser et al., 2017a | VPD | Stomatal opening | Tomato | 0.8, 1.5, 2.3 kPa | faster |
| Kaiser et al., 2017b | [CO_2_] | Photosynthetic induction | Tomato | 400, 800 ppm | faster |
| Kaiser et al., 2017b | [CO_2_] | Loss of photosynthetic induction | Tomato | 400, 800 ppm | slower |
| Kaiser et al., 2017b | [CO_2_] | Stomatal opening | Tomato | 400, 800 ppm | same |
| Kaiser et al., 2017b | [CO_2_] | Stomatal closure | Tomato | 400, 800 ppm | slower |
| Kaiser et al., 2020 | VPD | Photosynthetic induction | Tomato | 7, 15 mbar | slower |
| Kaiser et al., 2020 | VPD | Stomatal opening | Tomato | 7, 15 mbar | faster |
| Kang et al., 2020 | VPD x heat | Photosynthetic induction | *Croton argyratus* | 1.5 kPa x 30 °C; 3 kPa x 40 °C | slower |
| Kang et al., 2020 | VPD x heat | Photosynthetic induction | *Shorea leprosula* | 1.5 kPa x 30 °C; 3 kPa x 40 °C | slower |
| Kang et al., 2020 | VPD x heat | Photosynthetic induction | *Neobalanocarpus heimii* | 1.5 kPa x 30 °C; 3 kPa x 40 °C | same |
| Kang et al., 2020 | VPD x heat | Photosynthetic induction | *Lepisanthes senegalensis* | 1.5 kPa x 30 °C; 3 kPa x 40 °C | slower |
| Kang et al., 2020 | VPD x heat | Stomatal opening | *Croton argyratus* | 1.5 kPa x 30 °C; 3 kPa x 40 °C | slower |
| Kang et al., 2020 | VPD x heat | Stomatal opening | *Shorea leprosula* | 1.5 kPa x 30 °C; 3 kPa x 40 °C | same |
| Kang et al., 2020 | VPD x heat | Stomatal opening | *Neobalanocarpus heimii* | 1.5 kPa x 30 °C; 3 kPa x 40 °C | same |
| Kang et al., 2020 | VPD x heat | Stomatal opening | *Lepisanthes senegalensis* | 1.5 kPa x 30 °C; 3 kPa x 40 °C | same |
| Kang et al., 2021 | [CO_2_] | Photosynthetic induction | Rice | 400, 600 ppm | same |
| Kang et al., 2021 | [CO_2_] | Photosynthetic induction | Wheat | 400, 600 ppm | faster |
| Kang et al., 2021 | [CO_2_] | Stomatal opening | Rice | 400, 600 ppm | same |
| Kang et al., 2021 | [CO_2_] | Stomatal opening | Wheat | 400, 600 ppm | faster |
| Kang et al., 2021 | [CO_2_] | Post-illumination CO_2_ gain | Rice | 400, 600 ppm | larger |
| Kang et al., 2021 | [CO_2_] | Post-illumination CO_2_ gain | Wheat | 400, 600 ppm | same |
| Kueppers and Schneider, 1993 | Heat | Photosynthetic induction | *Fagus sylvatica* | 25, 35 °C | slower |
| Kueppers and Schneider, 1993 | Heat | Loss of photosynthetic induction | *Fagus sylvatica* | 25, 35 °C | faster |
| Leakey et al., 2002 | [CO_2_] | Photosynthetic induction | *Shorea leprosula* | 376, 711 ppm | faster |
| Leakey et al., 2002 | [CO_2_] | Stomatal opening | *Shorea leprosula* | 376, 711 ppm | slower |
| Leakey et al., 2002 | [CO_2_] | Post-illumination CO_2_ burst | *Shorea leprosula* | 376, 711 ppm | decrease |
| Leakey et al., 2002 | [CO_2_] | Post-illumination CO_2_ gain | *Shorea leprosula* | 376, 711 ppm | Increase |
| Leakey et al., 2002 | [CO_2_] | Loss of photosynthetic induction | *Shorea leprosula* | 376, 711 ppm | slower |
| Leakey et al., 2002 | [CO_2_] | Stomatal closure | *Shorea leprosula* | 376, 711 ppm | same |
| Leakey et al., 2003 | Heat | Photosynthetic induction | *Shorea leprosula* | 28, 38 °C | same |
| Leakey et al., 2003 | Heat | Stomatal opening | *Shorea leprosula* | 28, 38 °C | same |
| Leakey et al., 2003 | Heat | Loss of photosynthetic induction | *Shorea leprosula* | 28, 38 °C | faster |
| Leakey et al., 2003 | Heat | Stomatal closure | *Shorea leprosula* | 28, 38 °C | faster |
| Liu et al., 2025 | VPD | Photosynthetic induction | *Catalpa fargesii* | 1.3, 2.7 kPa | same |
| Liu et al., 2025 | VPD | Photosynthetic induction | *Pterocarya stenoptera* | 1.3, 2.7 kPa | slower |
| Liu et al., 2025 | VPD | Stomatal closure | *Catalpa fargesii* | 1.3, 2.7 kPa | same |
| Liu et al., 2025 | VPD | Stomatal closure | *Pterocarya stenoptera* | 1.3, 2.7 kPa | same |
| Liu et al., 2025 | VPD | Stomatal opening | *Catalpa fargesii* | 1.3, 2.7 kPa | faster |
| Liu et al., 2025 | VPD | Stomatal opening | *Pterocarya stenoptera* | 1.3, 2.7 kPa | same |
| Meng et al., 2026 | Drought | Photosynthetic induction | *Leymus chinensis* | 60-75, 35-50, 10-25 v/v | slower |
| Meng et al., 2026 | Drought | Stomatal opening | *Leymus chinensis* | 60-75, 35-50, 10-25 v/v | slower |
| Ozturk et al., 2012 | Heat | Photosynthetic induction | *Chrysanthemum morifolium* | 25, 30, 35 °C | faster |
| Qu et al., 2016 | Drought | Stomatal closure | Rice | 100, 8-12 v/v | faster |
| Qu et al., 2016 | Drought | Stomatal opening | Rice | 100, 8-12 v/v | same |
| Qu et al., 2020 | Drought | Stomatal closure | Rice | 100, 8-12 v/v | faster |
| Sakoda et al., 2022 | Drought | Photosynthetic induction | Rice | ? | same |
| Sakoda et al., 2022 | Drought | Photosynthetic induction | Soybean | ? | faster |
| Sakoda et al., 2022 | Drought | Stomatal opening | Rice | ? | faster |
| Sakoda et al., 2022 | Drought | Stomatal opening | Soybean | ? | faster |
| Shi et al., 2024 | VPD | Photosynthetic induction | Maize | 1.49, 3.61 kPa | same |
| Shi et al., 2024 | VPD | Photosynthetic induction | Tomato | 1.49, 3.61 kPa | slower |
| Shi et al., 2024 | VPD | Stomatal closure | Maize | 1.49, 3.61 kPa | faster |
| Shi et al., 2024 | VPD | Stomatal closure | Tomato | 1.49, 3.61 kPa | faster |
| Shi et al., 2024 | VPD | Stomatal opening | Maize | 1.49, 3.61 kPa | same |
| Shi et al., 2024 | VPD | Stomatal opening | Tomato | 1.49, 3.61 kPa | faster |
| Spormann et al., 2025 | Drought | Photosynthetic induction | Tomato | 100, 30% FC | same |
| Sun et al., 2023 | Drought | Photosynthetic induction | Tomato | 400, 200 g water pot^-1^ week^-1^ | slower |
| Sun et al., 2023 | Drought | Stomatal opening | Tomato | 400, 200 g water pot^-1^ week^-1^ | faster |
| Tinoco-Ojanguren and Pearcy, 1993a | VPD | Stomatal opening | *Piper auritum* | 0.6, 1.2, 1.8 kPa | faster |
| Tinoco-Ojanguren and Pearcy, 1993a | VPD | Stomatal opening | *Piper aequale* | 0.6, 1.2, 1.8 kPa | faster |
| Tinoco-Ojanguren and Pearcy, 1993b | VPD | Stomatal opening | *Piper auritum* | 0.6, 1.8 kPa | faster |
| Tinoco-Ojanguren and Pearcy, 1993b | VPD | Stomatal opening | *Piper aequale* | 0.6, 1.8 kPa | faster |
| Tinoco-Ojanguren and Pearcy, 1993b | VPD | Stomatal closure | *Piper auritum* | 0.6, 1.8 kPa | faster |
| Tinoco-Ojanguren and Pearcy, 1993b | VPD | Stomatal closure | *Piper aequale* | 0.6, 1.8 kPa | same |
| Tomimatsu and Tang, 2012 | [CO_2_] | Photosynthetic induction | *Populus koreana x trichocarpa* | 380, 700 ppm | faster |
| Tomimatsu and Tang, 2012 | [CO_2_] | Photosynthetic induction | *Populus euramericana* | 380, 700 ppm | faster |
| Tomimatsu and Tang, 2012 | [CO_2_] | Stomatal opening | *Populus koreana x trichocarpa* | 380, 700 ppm | faster |
| Tomimatsu and Tang, 2012 | [CO_2_] | Stomatal opening | *Populus euramericana* | 380, 700 ppm | same |
| Tomimatsu et al., 2014 | [CO_2_] | Photosynthetic induction | *Dipterocarpus sublamellatus* | 350, 700 ppm | faster |
| Tomimatsu et al., 2014 | [CO_2_] | Post-illumination CO_2_ gain | *Dipterocarpus sublamellatus* | 350, 700 ppm | Increase |
| Tomimatsu et al., 2014 | [CO_2_] | Post-illumination CO_2_ burst | *Dipterocarpus sublamellatus* | 350, 700 ppm | same |
| Wachendorf and Kueppers, 2017 | VPD x heat | Stomatal opening | *Betula pubescens* | 11.5 x 25, 20.8 x 30, 35.1 mmol mol^-1^ x 35 °C | faster |
| Wachendorf and Kueppers, 2017 | VPD x heat | Stomatal opening | *Fagus sylvatica* | 11.5 x 25, 20.8 x 30, 35.1 mmol mol^-1^ x 35 °C | faster |
| Wachendorf and Kueppers, 2017 | VPD x heat | Photosynthetic induction | *Betula pubescens* | 11.5 x 25, 20.8 x 30, 35.1 mmol mol^-1^ x 35 °C | faster |
| Wachendorf and Kueppers, 2017 | VPD x heat | Photosynthetic induction | *Fagus sylvatica* | 11.5 x 25, 20.8 x 30, 35.1 mmol mol^-1^ x 35 °C | faster |
| Yamori et al., 2014 | Heat | Photosynthetic induction | Rice | 25, 40 °C | faster |
| Zhang et al., 2024b | [CO_2_] | Photosynthetic induction | Chrysanthemum | 450, 900 ppm | faster |
| Zhang et al., 2024b | [CO_2_] | Photosynthetic induction | Tomato | 450, 900 ppm | same |
| Zhang et al., 2024b | [CO_2_] | Photosynthetic induction | Cucumber | 450, 900 ppm | same |
| Zhang et al., 2024b | [CO_2_] | Post-illumination CO_2_ gain | Chrysanthemum | 450, 900 ppm | same |
| Zhang et al., 2024b | [CO_2_] | Post-illumination CO_2_ gain | Tomato | 450, 900 ppm | same |
| Zhang et al., 2024b | [CO_2_] | Post-illumination CO_2_ gain | Cucumber | 450, 900 ppm | same |
| Zhang et al., 2024b | [CO_2_] | Stomatal opening | Chrysanthemum | 450, 900 ppm | same |
| Zhang et al., 2024b | [CO_2_] | Stomatal opening | Tomato | 450, 900 ppm | same |
| Zhang et al., 2024b | [CO_2_] | Stomatal opening | Cucumber | 450, 900 ppm | same |
| Zhang et al., 2024b | [CO_2_] | Stomatal closure | Chrysanthemum | 450, 900 ppm | same |
| Zhang et al., 2024b | [CO_2_] | Stomatal closure | Tomato | 450, 900 ppm | same |
| Zhang et al., 2024b | [CO_2_] | Stomatal closure | Cucumber | 450, 900 ppm | faster |

Table S8. Long-term effects (days-weeks) of environmental factors associated with global climate change on dynamic photosynthesis and its underlying processes. Processes include plant growth, the rate of photosynthetic induction, rates of stomatal opening and closure, and time-integrated post-illumination CO_2_ gain. Effects on a given process are stated based on an increase in a given environmental factor. Abbreviations: FL, fluctuating light; ppm, parts per million.

| **Study** | **Environmental factor** | **Process** | **Species** | **Factor levels** | **Effect on process** |
| --- | --- | --- | --- | --- | --- |
| Kang et al., 2021 | acclimation to CO_2_ | Post-illumination CO_2_ gain | Rice | 400, 600 ppm | increase |
| Kang et al., 2021 | acclimation to CO_2_ | Post-illumination CO_2_ gain | Wheat | 400, 600 ppm | decrease |
| Leakey et al., 2002 | FL x CO_2_ | Growth | Shorea leprosula | 376, 711 ppm | Increase |
| Shrestha et al., 2025 | FL x CO_2_ | Growth | Cucumber | 440, 860 ppm | increase |
| Shrestha et al., 2025 | acclimation to CO_2_ | Photosynthetic induction | Cucumber | 440, 860 ppm | same |
| Shrestha et al., 2025 | acclimation to CO_2_ | Stomatal opening | Cucumber | 440, 860 ppm | same |
| Shrestha et al., 2025 | acclimation to CO_2_ | Stomatal closure | Cucumber | 440, 860 ppm | same |
| Zhang et al., 2024b | acclimation to CO_2_ | Photosynthetic induction | Chrysanthemum | 450, 900 ppm | faster |
| Zhang et al., 2024b | acclimation to CO_2_ | Photosynthetic induction | Tomato | 450, 900 ppm | same |
| Zhang et al., 2024b | acclimation to CO_2_ | Photosynthetic induction | Cucumber | 450, 900 ppm | same |
| Zhang et al., 2024b | acclimation to CO_2_ | Post-illumination CO_2_ gain | Chrysanthemum | 450, 900 ppm | decrease |
| Zhang et al., 2024b | acclimation to CO_2_ | Post-illumination CO_2_ gain | Tomato | 450, 900 ppm | same |
| Zhang et al., 2024b | acclimation to CO_2_ | Post-illumination CO_2_ gain | Cucumber | 450, 900 ppm | same |
| Zhang et al., 2024b | acclimation to CO_2_ | Stomatal opening | Chrysanthemum | 450, 900 ppm | same |
| Zhang et al., 2024b | acclimation to CO_2_ | Stomatal opening | Tomato | 450, 900 ppm | faster |
| Zhang et al., 2024b | acclimation to CO_2_ | Stomatal opening | Cucumber | 450, 900 ppm | same |
| Zhang et al., 2024b | acclimation to CO_2_ | Stomatal closure | Chrysanthemum | 450, 900 ppm | same |
| Zhang et al., 2024b | acclimation to CO_2_ | Stomatal closure | Tomato | 450, 900 ppm | same |
| Zhang et al., 2024b | acclimation to CO_2_ | Stomatal closure | Cucumber | 450, 900 ppm | faster |

References / Data sources

Adachi, S., Tanaka, Y., Miyagi, A., Kashima, M., Tezuka, A., Toya, Y., Kobayashi, S., Ohkubo, S., Shimizu, H., Kawai-Yamada, M., Sage, R. F., Nagano, A. J., & Yamori, W. (2019). High-yielding rice Takanari has superior photosynthetic response to a commercial rice Koshihikari under fluctuating light. *Journal of Experimental Botany*, *70*(19), 5287–5297. https://doi.org/10.1093/jxb/erz304

Alter, P., Dreissen, A., Luo, F. L., & Matsubara, S. (2012). Acclimatory responses of Arabidopsis to fluctuating light environment: Comparison of different sunfleck regimes and accessions. *Photosynthesis Research*, *113*(1–3), 221–237. https://doi.org/10.1007/s11120-012-9757-2

Ariura, R., Matsumoto, M., Li, J., Fuse, T., Aoki, T., Zhang, Y., Kinose, Y., Yamaguchi, M., Izuta, T., & Watanabe, M. (2023). Effects of elevated ozone and carbon dioxide on the dynamic photosynthesis of Fagus crenata seedlings under variable light conditions. *Science of The Total Environment*, *891*, 164398. https://doi.org/10.1016/j.scitotenv.2023.164398

Barradas, V. L., & Jones, H. G. (1996). Responses of CO2 assimilation to changes in irradiance: laboratory and field data and a model for beans [Phaseolus vulgaris L.). *Journal of Experimental Botany*, *47*(298), 639–645. http://jxb.oxfordjournals.org/

Barradas, V. L., Jones, H. G., & Clark, J. A. (1998). Sunfleck dynamics and canopy structure in a Phaseolus vulgaris L. canopy. *Int. J. Biometeorol.*, *42*, 34–43.

Bellasio, C. (2019). A generalised dynamic model of leaf-level C3 photosynthesis combining light and dark reactions with stomatal behaviour. *Photosynthesis Research*, *141*(1), 99–118. https://doi.org/10.1007/s11120-018-0601-1

Bhuiyan, R., & van Iersel, M. W. (2021). Only extreme fluctuations in light levels reduce lettuce growth under sole source lighting. *Frontiers in Plant Science*, *12*. https://doi.org/10.3389/fpls.2021.619973

Blom, T. J., & Zheng, Y. (2009). The response of plant growth and leaf gas exchange to the speed of lamp movement in a greenhouse. *Scientia Horticulturae*, *119*(2), 188–192. https://doi.org/10.1016/j.scienta.2008.07.014

Burgess, A. J., Durand, M., Gibbs, J. A., Retkute, R., Robson, T. M., & Murchie, E. H. (2021). The effect of canopy architecture on the patterning of “windflecks” within a wheat canopy. *Plant, Cell & Environment*, *44*(11), 3524–3537. https://doi.org/10.1111/pce.14168

Burgess, A. J., Retkute, R., & Murchie, E. H. (2023). Photoacclimation and entrainment of photosynthesis by fluctuating light varies according to genotype in Arabidopsis thaliana. *Frontiers in Plant Science*, *14*. https://doi.org/10.3389/fpls.2023.1116367

Caine, R. S., Khan, M. S., Shan, Y., Osborne, C. P., & Croft, H. L. (2026). Future heatwave conditions inhibit <scp> CO _2_ </scp> ‐induced stomatal closure in wheat. *New Phytologist*, *249*(3), 1234–1252. https://doi.org/10.1111/nph.70722

Carmo-Silva, A. E., & Salvucci, M. E. (2013). The Regulatory Properties of Rubisco Activase Differ among Species and Affect Photosynthetic Induction during Light Transitions    . *Plant Physiology*, *161*(4), 1645–1655. https://doi.org/10.1104/pp.112.213348

Chazdon, R. L., & Pearcy, R. W. (1986). Photosynthetic responses to light variation in rainforest species I. Induction under constant and fluctuating light conditions. *Oecologia*, *69*, 517–523.

Clemente, H. S., & Marler, T. E. (1996). Drought Stress Influences Gas-exchange Responses of Papaya Leaves to Rapid Changes in Irradiance. *J. Amer. Soc. Hort. Sci.*, *121*(2), 292–295.

Dieleman, J. A., van Steekelenburg, G., Weerheim, K., Kaiser, E., Meinen, E., & van Hoogdalem, M. (2025). Frequency of light fluctuations affects tomato morphology and physiology only at extreme amplitudes. *Frontiers in Plant Science*, *16*. https://doi.org/10.3389/fpls.2025.1500197

Ding, J., Zhao, J., Pan, T., Xi, L., Zhang, J., & Zou, Z. (2019). Comparative Transcriptome Analysis of Gene Expression Patterns in Tomato Under Dynamic Light Conditions. *Genes*, *10*(9), 662. https://doi.org/10.3390/genes10090662

Doehlert, D. C., Ku, M. S. B., & Edwards, G. E. (1979). Dependence of the post-illumination burst of CO2 on Temperature, Light, CO2, and Concentration in Wheat (Triticum aestivum). *Physiologia Plantarum*, *46*, 299–306.

Dokoozlian, N. K., & Kliewer, W. M. (1995). The Light Environment Within Grapevine Canopies. I. Description and Seasonal Changes During Fruit Development. *Am. J. Enol. Vitic.*, *42*(2), 209–218.

Durand, M., Gibbs, J. A., Murchie, E. H., Robson, T. M., & Gibbs, A. J. (2026). When wuthering winds create fluttering fields: structural and biomechanical properties determine canopy light fluctuation properties of 10 wheat cultivars. *New Phytologist*. https://doi.org/10.1111/nph.70975

Durand, M., Matule, B., Burgess, A. J., & Robson, T. M. (2021). Sunfleck properties from time series of fluctuating light. *Agricultural and Forest Meteorology*, *308–309*. https://doi.org/10.1016/j.agrformet.2021.108554

Durand, M., & Robson, T. M. (2023). Fields of a thousand shimmers: canopy architecture determines high-frequency light fluctuations. *New Phytologist*, *238*(5), 2000–2015. https://doi.org/10.1111/nph.18822

Durand, M., Zhuang, X., Salmon, Y., & Robson, T. M. (2024). Caught between two states: The compromise in acclimation of photosynthesis, transpiration and mesophyll conductance to different amplitudes of fluctuating irradiance. *Plant, Cell & Environment*, *47*(12), 5220–5236. https://doi.org/10.1111/pce.15107

Fan, Y., Hussain, S., Brestic, M., Xiang, C., & Yang, F. (2025). Differential Photosynthetic Acclimation to Fluctuating Light Reveals Specific Photoprotective Strategies in Shade‐Tolerant Soybean. *Physiologia Plantarum*, *177*(5). https://doi.org/10.1111/ppl.70554

Ferroni, L., Živčak, M., Sytar, O., Kovár, M., Watanabe, N., Pancaldi, S., Baldisserotto, C., & Brestič, M. (2020). Chlorophyll-depleted wheat mutants are disturbed in photosynthetic electron flow regulation but can retain an acclimation ability to a fluctuating light regime. *Environmental and Experimental Botany*, *178*, 104156. https://doi.org/10.1016/j.envexpbot.2020.104156

Garcia-Molina, A., & Leister, D. (2020). Accelerated relaxation of photoprotection impairs biomass accumulation in Arabidopsis. *Nature Plants*, *6*(1), 9–12. https://doi.org/10.1038/s41477-019-0572-z

Gross, L. J. (1982). Photosynthetic Dynamics in Varying Light Environments: A Model and Its Application to Whole Leaf Carbon Gain. *Ecology*, *63*(1), 84–93.

Gross, L. J., Kirschbaum, M. U. F., & Pearcy, R. W. (1991). A dynamic model of photosynthesis in varying light taking account of stomatal conductance, C3-cycle intermediates, photorespiration and Rubisco activation. *Plant, Cell and Environment*, *14*, 881–893.

Gutschick, V. P., Barron, M. H., Waechter, D. A., & Wolf, M. A. (1985). Portable monitor for solar radiation that accumulates irradiance histograms for 32 leaf-mounted sensors. *Agricultural and Forest Meteorology*, *33*(4), 281–290. https://doi.org/10.1016/0168-1923(85)90028-0

Hermida-Carrera, C., Kapralov, M. V., & Galmés, J. (2016). Rubisco Catalytic Properties and Temperature Response in Crops. *Plant Physiology*, *171*(4), 2549–2561. https://doi.org/10.1104/pp.16.01846

Heuermann, M. C., Meyer, R. C., Knoch, D., Tschiersch, H., & Altmann, T. (2024). Strong prevalence of light regime‐specific <scp>QTL</scp> in Arabidopsis detected using automated high‐throughput phenotyping in fluctuating or constant light. *Physiologia Plantarum*, *176*(2). https://doi.org/10.1111/ppl.14255

Huang, G., Zhang, Q., Wei, X., Peng, S., & Li, Y. (2017). Nitrogen Can Alleviate the Inhibition of Photosynthesis Caused by High Temperature Stress under Both Steady-State and Flecked Irradiance. *Frontiers in Plant Science*, *8*. https://doi.org/10.3389/fpls.2017.00945

Hubbart, S., Smillie, I. R. A., Heatley, M., Swarup, R., Foo, C. C., Zhao, L., & Murchie, E. H. (2018). Enhanced thylakoid photoprotection can increase yield and canopy radiation use efficiency in rice. *Communications Biology*, *1*(1), 22. https://doi.org/10.1038/s42003-018-0026-6

Impens, I., Lemeur, R., & Moermans, R. (1970). Spatial and temporal variation of net radiation in crop canopies. *Agricultural Meteorology*, *7*, 335–337.

Jezek, M., Silva-Alvim, F. A. L., Hills, A., Donald, N., Ishka, M. R., Shadbolt, J., He, B., Lawson, T., Harper, J. F., Wang, Y., Lew, V. L., & Blatt, M. R. (2021). Guard cell endomembrane Ca2+-ATPases underpin a ‘carbon memory’ of photosynthetic assimilation that impacts on water-use efficiency. *Nature Plants*, *7*(9), 1301–1313. https://doi.org/10.1038/s41477-021-00966-2

Jishi, T., Matsuda, R., & Fujiwara, K. (2015). A kinetic model for estimating net photosynthetic rates of cos lettuce leaves under pulsed light. *Photosynthesis Research*, *124*(1), 107–116. https://doi.org/10.1007/s11120-015-0107-z

Joubert, D., Zhang, N., Berman, Sarah. R., Kaiser, E., Molenaar, J., & Stigter, J. D. (2023). A small dynamic leaf-level model predicting photosynthesis in greenhouse tomatoes. *PLOS ONE*, *18*(3), e0275047. https://doi.org/10.1371/journal.pone.0275047

Kaiser, E., Kromdijk, J., Harbinson, J., Heuvelink, E., & Marcelis, L. F. M. (2017). Photosynthetic induction and its diffusional, carboxylation and electron transport processes as affected by CO<inf>2</inf>partial pressure, temperature, air humidity and blue irradiance. *Annals of Botany*, *119*(1). https://doi.org/10.1093/aob/mcw226

Kaiser, E., Kusuma, P., Vialet-Chabrand, S., Folta, K., Liu, Y., Poorter, H., Woning, N., Shrestha, S., Ciarreta, A., van Brenk, J., Karpe, M., Ji, Y., David, S., Zepeda, C., Zhu, X.-G., Huntenburg, K., Verdonk, J. C., Woltering, E., Gauthier, P. P. G., … Marcelis, L. F. M. (2024). Vertical farming goes dynamic: optimizing resource use efficiency, product quality, and energy costs. *Frontiers in Science*, *2*. https://doi.org/10.3389/fsci.2024.1411259

Kaiser, E., Matsubara, S., Harbinson, J., Heuvelink, E., & Marcelis, L. F. M. (2018). Acclimation of photosynthesis to lightflecks in tomato leaves: interaction with progressive shading in a growing canopy. *Physiologia Plantarum*, *162*(4). https://doi.org/10.1111/ppl.12668

Kaiser, E., Morales, A., & Harbinson, J. (2018). Fluctuating light takes crop photosynthesis on a rollercoaster ride. *Plant Physiology*, *176*(2). https://doi.org/10.1104/pp.17.01250

Kaiser, E., Morales, A., Harbinson, J., Heuvelink, E., & Marcelis, L. F. M. (2020). High stomatal conductance in the tomato Flacca mutant allows for faster photosynthetic induction. *Frontiers in Plant Science*, *11*(August), 1–12. https://doi.org/10.3389/fpls.2020.01317

Kaiser, E., Zhou, D., Heuvelink, E., Harbinson, J., Morales, A., & Marcelis, L. F. M. (2017). Elevated CO_2_ increases photosynthesis in fluctuating irradiance regardless of photosynthetic induction state. *Journal of Experimental Botany*, *68*(20), 5629–5640. https://doi.org/10.1093/jxb/erx357

Kang, H., Zhu, T., Zhang, Y., Ke, X., Sun, W., Hu, Z., Zhu, X., Shen, H., Huang, Y., & Tang, Y. (2021). Elevated CO2 Enhances Dynamic Photosynthesis in Rice and Wheat. *Frontiers in Plant Science*, *12*. https://doi.org/10.3389/fpls.2021.727374

Kang, H.-X., Zhu, X.-G., Yamori, W., & Tang, Y.-H. (2020). Concurrent Increases in Leaf Temperature With Light Accelerate Photosynthetic Induction in Tropical Tree Seedlings. *Frontiers in Plant Science*, *11*. https://doi.org/10.3389/fpls.2020.01216

Kimura, H., Hashimoto-Sugimoto, M., Iba, K., Terashima, I., & Yamori, W. (2020). Improved stomatal opening enhances photosynthetic rate and biomass production in fluctuating light. *Journal of Experimental Botany*, *71*(7), 2339–2350. https://doi.org/10.1093/jxb/eraa090

Kirschbaum, M. U. F., & Farquhar, G. D. (1984). Temperature Dependence of Whole-Leaf Photosynthesis in *Eucalyptus pauciflora* Sieb. Ex Spreng. *Australian Journal of Plant Physiology*, *11*(6), 519–538. https://doi.org/10.1071/PP9840519

Kirschbaum, M. U. F., Kueppers, M., Schneider, H., Giersch, C., & Noe, S. (1998). Modelling photosynthesis in fluctuating light with inclusion of stomatal conductance, biochemical activation and pools of key photosynthetic intermediates. *Planta*, *204*, 16–26.

Kubásek, J., Urban, O., & Šantrůček, J. (2013). C4 plants use fluctuating light less efficiently than do C3 plants: a study of growth, photosynthesis and carbon isotope discrimination. *Physiologia Plantarum*, *149*(4), 528–539. https://doi.org/10.1111/ppl.12057

Kueppers M., & Schneider H. (1993). Leaf gas exchange of beech (Fagus sylvatica L.) seedlings in lightflecks: effects of fleck length and leaf temperature in leaves grown in deep and partial shade. *Trees*, *7*, 160–168.

Laisk, A., Eichelmann, H., & Oja, V. (2006). C3 photosynthesis in silico. *Photosynthesis Research*, *90*(1), 45–66. https://doi.org/10.1007/s11120-006-9109-1

Lakso, A. N., & Barnes, J. E. (1978). Apple Leaf Photosynthesis in Alternating Light. *HortScience*, *13*(4), 473–474.

Leakey, A. D. B., Press, M. C., & Scholes, J. D. (2003). High-temperature inhibition of photosynthesis is greater under sunflecks than uniform irradiance in a tropical rain forest tree seedling. *Plant, Cell and Environment*, *26*(10), 1681–1690. https://doi.org/10.1046/j.1365-3040.2003.01086.x

Leakey, A. D. B., Press, M. C., Scholes, J. D., & Watling, J. R. (2002). Relative enhancement of photosynthesis and growth at elevated CO2 is greater under sunflecks than uniform irradiance in a tropical rain forest tree seedling. *Plant, Cell and Environment*, *25*(12), 1701–1714. https://doi.org/10.1046/j.1365-3040.2002.00944.x

Lei, Z., Li, X., Li, Y., Zhang, T., Li, X., Yang, Y., Zhang, Y., & He, D. (2025). Photosynthetic mechanism of cotton under fluctuating light field planted with different densities. *Industrial Crops and Products*, *228*, 120920. https://doi.org/10.1016/j.indcrop.2025.120920

Li, X., Li, X., Zhang, T., Xue, X., Dai, Y., Lei, Z., & He, D. (2025). High plant density optimizes leaf stomatal traits for accelerating the stomatal response rate at the lower cotton canopy. *Crop Science*, *65*(1). https://doi.org/10.1002/csc2.21443

Liu, N., Cao, J., Yang, M., Li, Y., & Huang, W. (2025). The Kinetics of Mesophyll Conductance and Photorespiration During Light Induction. *Plants*, *14*(6), 850. https://doi.org/10.3390/plants14060850

Makino, A., Mae, T., & Ohira, K. (1988). Differences between wheat and rice in the enzymic properties of ribulose-1,5-bisphosphate carboxylase/oxygenase and the relationship to photosynthetic gas exchange. *Planta*, *174*(1), 30–38. https://doi.org/10.1007/BF00394870

Matsuda, R., Shiba, S., Chen, Y., Kubo, S., Yu, L., Lee, J.-Y., & Fujiwara, K. (2023). Growth of cucumber seedlings under emulated sunlight with artificially reproduced fluctuations in photosynthetic photon flux density. *Journal of Agricultural Meteorology*, *79*(4), D-23-00019. https://doi.org/10.2480/agrmet.D-23-00019

Meng, D., Xu, R., Cao, L., Ma, J., & Sun, W. (2025). Photosynthetic induction responses of two Leymus chinensis ecotypes under drought stress. *BMC Plant Biology*, *26*(1), 137. https://doi.org/10.1186/s12870-025-07982-w

Morales, A., Kaiser, E., Yin, X., Harbinson, J., Molenaar, J., Driever, S. M., & Struik, P. C. (2018). Dynamic modelling of limitations on improving leaf CO2 assimilation under fluctuating irradiance. *Plant, Cell & Environment*, *41*, 589–604. https://doi.org/10.1111/pce.13119

Mott, K. A., & Woodrow, I. E. (2000). Modelling the role of Rubisco activase in limiting non-steady-state photosynthesis. *Journal of Experimental Botany*, *51*, 399–406.

Naumburg, E., & Ellsworth, D. S. (2002). Short-term light and leaf photosynthetic dynamics affect estimates of daily understory photosynthesis in four tree species. *Tree Physiology*, *22*(6), 393–401. https://doi.org/10.1093/treephys/22.6.393

Naumburg, E., Ellsworth, D. S., & Katul, G. G. (2001). Modeling dynamic understory photosynthesis of contrasting species in ambient and elevated carbon dioxide. *Oecologia*, *126*(4), 487–499. https://doi.org/10.1007/s004420000543

Nishimura, S., Koizumi, H., & Tang, Y. (1998). Spatial and Temporal Variation in Photon Flux Density on Rice ( *Oryza sativa* L.) Leaf Surface. *Plant Production Science*, *1*(1), 30–36. https://doi.org/10.1626/pps.1.30

Noe, S. M., & Giersch, C. (2004). A simple dynamic model of photosynthesis in oak leaves: Coupling leaf conductance and photosynthetic carbon fixation by a variable intracellular CO2 pool. *Functional Plant Biology*, *31*(12), 1195–1204. https://doi.org/10.1071/FP03251

Ooba, M., Takagi, K., & Takahashi, H. (2003). Numerical simulation of the time-averaged rate of photosynthesis in a leaf under alternating light. *J. Agric. Meteorol.*, *59*(4), 259–267.

Ozturk, I., Holst, N., & Ottosen, C.-O. (2012). Simulation of leaf photosynthesis of C3 plants under fluctuating light and different temperatures. *Acta Physiologiae Plantarum*, *34*(6), 2319–2329. https://doi.org/10.1007/s11738-012-1033-8

Ozturk, I., Ottosen, C.-O., & Ritz, C. (2013). The effect of temperature on photosynthetic induction under fluctuating light in Chrysanthemum morifolium. *Acta Physiologiae Plantarum*, *35*(4), 1179–1188. https://doi.org/10.1007/s11738-012-1157-x

Pearcy, R. W., Gross, L. J., & He, D. (1997). An improved dynamic model of photosynthesis for estimation of carbon gain in sunfleck light regimes. *Plant, Cell and Environment*, *20*, 411–424.

Pearcy, R. W., Roden, J. S., & Gamon, J. A. (1990). Sunfleck dynamics in relation to canopy structure in a soybean (Glycine max (L.) Merr. ) canopy. *Agricultural and Forest Meteorology*, *52*, 359–372.

Peressotti, A., Marchiol, L., & Zerbi, G. (2001). Photosynthetic photon flux density and sunfleck regime within canopies of wheat, sunflower and maize in different wind conditions. *Italian Journal of Agronomy*, *4*(2), 87–92.

Peri, P. L., Moot, D. J., & McNeil, D. L. (2006). Validation of a canopy photosynthesis model for cocksfoot pastures grown under different light regimes. *Agroforestry Systems*, *67*(3), 259–272. https://doi.org/10.1007/s10457-005-3825-y

Porcar-Castell, A., Bäck, J., Juurola, E., & Hari, P. (2006). Dynamics of the energy flow through photosystem II under changing light conditions: A model approach. *Functional Plant Biology*, *33*(3), 229–239. https://doi.org/10.1071/FP05133

Qiao, M.-Y., Zhang, Y.-J., Liu, L.-A., Shi, L., Ma, Q.-H., Chow, W. S., & Jiang, C.-D. (2021). Do rapid photosynthetic responses protect maize leaves against photoinhibition under fluctuating light? *Photosynthesis Research*, *149*(1–2), 57–68. https://doi.org/10.1007/s11120-020-00780-5

Qu, M., Essemine, J., Xu, J., Ablat, G., Perveen, S., Wang, H., Chen, K., Zhao, Y., Chen, G., Chu, C., & Zhu, X. (2020). Alterations in stomatal response to fluctuating light increase biomass and yield of rice under drought conditions. *The Plant Journal*, *104*(5), 1334–1347. https://doi.org/10.1111/tpj.15004

Qu, M., Hamdani, S., Li, W., Wang, S., Tang, J., Chen, Z., Song, Q., Li, M., Zhao, H., Chang, T., Chu, C., & Zhu, X. (2016). Rapid stomatal response to fluctuating light: An under-explored mechanism to improve drought tolerance in rice. *Functional Plant Biology*, *43*(8), 727–738. https://doi.org/10.1071/FP15348

Roden, J. S. (2003). Modeling the light interception and carbon gain of individual fluttering aspen (Populus tremuloides Michx) leaves. *Trees*, *17*(2), 117–126. https://doi.org/10.1007/s00468-002-0213-3

Sakoda, K., Taniyoshi, K., Yamori, W., & Tanaka, Y. (2022). Drought stress reduces crop carbon gain due to delayed photosynthetic induction under fluctuating light conditions. *Physiologia Plantarum*, *174*(1). https://doi.org/10.1111/ppl.13603

Sakoda, K., Yamori, W., Shimada, T., Sugano, S. S., Hara-Nishimura, I., & Tanaka, Y. (2020). Higher Stomatal Density Improves Photosynthetic Induction and Biomass Production in Arabidopsis Under Fluctuating Light. *Frontiers in Plant Science*, *11*. https://doi.org/10.3389/fpls.2020.589603

Salter, W. T., Merchant, A. M., Richards, R. A., Trethowan, R., & Buckley, T. N. (2019). Rate of photosynthetic induction in fluctuating light varies widely among genotypes of wheat. *Journal of Experimental Botany*, *70*(10), 2787–2796. https://doi.org/10.1093/jxb/erz100

Salvatori, N., Alberti, G., Muller, O., & Peressotti, A. (2022). Does Fluctuating Light Affect Crop Yield? A Focus on the Dynamic Photosynthesis of Two Soybean Varieties. *Frontiers in Plant Science*, *13*. https://doi.org/10.3389/fpls.2022.862275

Salvatori, N., Carteni, F., Giannino, F., Alberti, G., Mazzoleni, S., & Peressotti, A. (2022). A System Dynamics Approach to Model Photosynthesis at Leaf Level Under Fluctuating Light. *Frontiers in Plant Science*, *12*. https://doi.org/10.3389/fpls.2021.787877

Sassenrath-Cole, G. F., & Pearcy, R. W. (1994). Regulation of Photosynthetic Induction State by the Magnitude and Duration of Low Light Exposure. *Plant Physiology*, *105*(4), 1115–1123. https://doi.org/10.1104/pp.105.4.1115

Shao, B., Zhang, Y., Vincenzi, E., Berman, S., Vialet-Chabrand, S., Marcelis, L. F. M., Li, T., & Kaiser, E. (2024). Photosynthesis and photoprotection in top leaves respond faster to irradiance fluctuations than bottom leaves in a tomato canopy. *Journal of Experimental Botany*, *75*(22), 7217–7236. https://doi.org/10.1093/jxb/erae357

Shao, M., Liu, W., Zha, L., Zhou, C., Zhang, Y., & Li, B. (2020). Effects of constant versus fluctuating red–blue LED radiation on yield and quality of hydroponic purple-leaf lettuce. *Horticulture, Environment, and Biotechnology*, *61*(6), 989–997. https://doi.org/10.1007/s13580-020-00285-z

Shi, Q., Wang, X., He, B., Yang, Y., & Huang, W. (2024). Differential impact of decreasing relative humidity on photosynthesis under fluctuating light between maize and tomato. *Physiologia Plantarum*, *176*(1). https://doi.org/10.1111/ppl.14179

Shimadzu, S., Seo, M., Terashima, I., & Yamori, W. (2019). Whole Irradiated Plant Leaves Showed Faster Photosynthetic Induction Than Individually Irradiated Leaves via Improved Stomatal Opening. *Frontiers in Plant Science*, *10*. https://doi.org/10.3389/fpls.2019.01512

Shrestha, S., Berman, S. R., Oosterkamp, J., Marcelis, L. F. M., Kaiser, E., & Vialet‐Chabrand, S. (2025). Elevated [CO_2_] Affected Fluctuating Light Acclimation in Cucumber Plants by Changes in Specific Leaf Area and Photosynthetic Efficiency. *Physiologia Plantarum*, *177*(4). https://doi.org/10.1111/ppl.70436

Sikma, M., Ikawa, H., Heusinkveld, B. G., Yoshimoto, M., Hasegawa, T., Groot Haar, L. T., Anten, N. P. R., Nakamura, H., Vilà‐Guerau de Arellano, J., Sakai, H., Tokida, T., Usui, Y., & Evers, J. B. (2020). Quantifying the Feedback Between Rice Architecture, Physiology, and Microclimate Under Current and Future CO _2_ Conditions. *Journal of Geophysical Research: Biogeosciences*, *125*(3). https://doi.org/10.1029/2019JG005452

Sims, D. A., & Pearcy, R. W. (1993). Sunfleck Frequency and Duration Affects Growth Rate of the  understorey plant, Alocasia macrorrhiza. *Functional Ecology*, *7*(6), 683–689.

Spormann, S., Neves, J., Pereira, C., Soares, C., Valente, I. M., Rodrigues, J. A., Martins, V., Kaiser, E., & Fidalgo, F. (2026). Dissecting the physiology of wild tomatoes under abiotic stress: Dynamic photosynthesis and metabolic adaptations to combined drought and salinity. *Journal of Plant Physiology*, *316*, 154664. https://doi.org/10.1016/j.jplph.2025.154664

Stegemann, J., Timm, H.-C., & Küppers, M. (1999). Simulation of photosynthetic plasticity in response to highly fluctuating light: an empirical model integrating dynamic photosynthetic induction and capacity. *Trees*, *14*, 145–160.

Stinziano, J., Roback, C., Sargent, D., Murphy, B., Hudson, P., & Muir, P. (2024). *photosynthesis: Tools for Plant Ecophysiology & Modeling (Version 2.1.5)*. https://cran.r-project.org/web/packages/photosynthesis/index.html

Sun, H., Shi, Q., Liu, N.-Y., Zhang, S.-B., & Huang, W. (2023). Drought stress delays photosynthetic induction and accelerates photoinhibition under short-term fluctuating light in tomato. *Plant Physiology and Biochemistry*, *196*, 152–161. https://doi.org/10.1016/j.plaphy.2023.01.044

Tang, Y.-H., Washitani, I., Tsushiya, T., & Iwaki, H. (1988). Fluctuation of Photosynthetic Photon Flux Density within a Miscanthus sinensis Canopy. *Ecol. Res*, *3*, 253–266.

Tatsumi, K., Kuwabara, Y., & Motobayashi, T. (2020). Photosynthetic light‐use efficiency of rice leaves under fluctuating incident light. *Agrosystems, Geosciences & Environment*, *3*(1). https://doi.org/10.1002/agg2.20030

Taylor, S. H., & Long, S. P. (2017). Slow induction of photosynthesis on shade to sun transitions in wheat may cost at least 21% of productivity. *Philosophical Transactions of the Royal Society B*, *372*, 1–9.

Thormählen, I., Zupok, A., Rescher, J., Leger, J., Weissenberger, S., Groysman, J., Orwat, A., Chatel-Innocenti, G., Issakidis-Bourguet, E., Armbruster, U., & Geigenberger, P. (2017). Thioredoxins Play a Crucial Role in Dynamic Acclimation of Photosynthesis in Fluctuating Light. *Molecular Plant*, *10*(1), 168–182. https://doi.org/10.1016/j.molp.2016.11.012

Timm, H.-C., Küppers, M., & Stegemann, J. (2004). Non-destructive analysis of architectural expansion and assimilate allocation in different tropical tree saplings: consequences of using steady-state and dynamic photosynthesis models. *Ecotropica*, *10*, 101–121.

Tinoco-Ojanguren, C., & Pearcy, R. W. (1993a). Stomatal Dynamics and Its Importance to Carbon Gain in Two Rainforest Piper Species. I. VPD Effects on the Transient Stomatal Response to Lightflecks. *Oecologia*, *94*, 388–394.

Tinoco-Ojanguren, C., & Pearcy, R. W. (1993b). Stomatal dynamics and its importance to carbon gain in two rainforest Piper species. II. Stomatal versus biochemical limitations during photosynthetic induction. *Oecologia*, *94*, 395–402.

Tomimatsu, H., Iio, A., Adachi, M., Saw, L.-G., Fletcher, C., & Tang, Y. (2014). High CO2 concentration increases relative leaf carbon gain under dynamic light in Dipterocarpus sublamellatus seedlings in a tropical rain forest, Malaysia. *Tree Physiology*, *00*, 1–11. https://doi.org/10.1093/treephys/tpu066.indd

Tomimatsu, H., & Tang, Y. (2012). Elevated CO2 differentially affects photosynthetic induction response in two Populus species with different stomatal behavior. *Oecologia*, *169*(4), 869–878. https://doi.org/10.1007/s00442-012-2256-5

Tong, H., & Hipps, L. E. (1996). The effect of turbulence on the light environment of alfalfa. *Agricultural and Forest Meteorology*, *80*, 249–261.

Vialet-Chabrand, S., Matthews, J. S. A., Brendel, O., Blatt, M. R., Wang, Y., Hills, A., Griffiths, H., Rogers, S., & Lawson, T. (2016). Modelling water use efficiency in a dynamic environment: An example using *Arabidopsis thaliana*. *Plant Science*, *251*, 65–74. https://doi.org/10.1016/j.plantsci.2016.06.016

Vialet-Chabrand, S., Matthews, J. S. A., Simkin, A. J., Raines, C. A., & Lawson, T. (2017). Importance of fluctuations in light on plant photosynthetic acclimation. *Plant Physiology*, *173*(4), 2163–2179. https://doi.org/10.1104/pp.16.01767

Vico, G., Manzoni, S., Palmroth, S., & Katul, G. (2011). Effects of stomatal delays on the economics of leaf gas exchange under intermittent light regimes. *New Phytologist*, *192*(3), 640–652. https://doi.org/10.1111/j.1469-8137.2011.03847.x

Wachendorf, M., & Küppers, M. (2017). Effects of leaf temperature on initial stomatal opening and their roles in overall and biochemical photosynthetic induction. *Trees*, *31*(5), 1667–1681. https://doi.org/10.1007/s00468-017-1577-8

Wang, Y., Burgess, S. J., de Becker, E. M., & Long, S. P. (2020). Photosynthesis in the fleeting shadows: an overlooked opportunity for increasing crop productivity? *The Plant Journal*, *101*(4), 874–884. https://doi.org/10.1111/tpj.14663

Wang, Y., Xing Chan, K., & Long, S. P. (2021). Towards a dynamic photosynthesis model to guide yield improvement in C4 crops. *Plant Journal*, *107*(2), 341–342. https://doi.org/10.1111/tpj.15408

Wang, Y., & Zhu, X.-G. (2025). A practical guide to using a kinetic model for improving the efficiency of C4 photosynthetic metabolism. *In Silico Plants*, *7*(2). https://doi.org/10.1093/insilicoplants/diaf010

Watling, J. R., Ball, M. C., & Woodrow, I. E. (1997). The utilization of lightflecks for growth in four australian rain-forest species. *Functional Ecology*, *11*(2), 231–239. https://doi.org/10.1046/j.1365-2435.1997.00073.x

Woodford, R., Watkins, J., Moore, M., Nix, S. J., Yee, S., Chan, K. X., Pogson, B., von Caemmerer, S., Furbank, R. T., & Ermakova, M. (2025). PROTON GRADIENT REGULATION 5 enables efficient C4 photosynthesis under fluctuating light. *Plant Physiology*, *199*(2). https://doi.org/10.1093/plphys/kiaf466

Wu, H.-Y., Qiao, M.-Y., Zhang, Y.-J., Kang, W.-J., Ma, Q.-H., Gao, H.-Y., Zhang, W.-F., & Jiang, C.-D. (2023). Photosynthetic mechanism of maize yield under fluctuating light environments in the field. *Plant Physiology*, *191*(2), 957–973. https://doi.org/10.1093/plphys/kiac542

Yamori, W., Kusumi, K., Iba, K., & Terashima, I. (2020). Increased stomatal conductance induces rapid changes to photosynthetic rate in response to naturally fluctuating light conditions in rice. *Plant, Cell and Environment*, *43*, 1230–1240. https://doi.org/10.1111/pce.13725

Yamori, W., Masumoto, C., Fukayama, H., & Makino, A. (2012). Rubisco activase is a key regulator of non‐steady‐state photosynthesis at any leaf temperature and, to a lesser extent, of steady‐state photosynthesis at high temperature. *The Plant Journal*, *71*(6), 871–880. https://doi.org/10.1111/j.1365-313X.2012.05041.x

Zhang, N., Berman, S. R., van den Berg, T., Chen, Y., Marcelis, L. F. M., & Kaiser, E. (2024). Biochemical versus stomatal acclimation of dynamic photosynthetic gas exchange to elevated CO _2_ in three horticultural species with contrasting stomatal morphology. *Plant, Cell & Environment*, *47*(12), 4516–4529. https://doi.org/10.1111/pce.15043

Zhang, Y., Kaiser, E., Marcelis, L. F. M., Yang, Q., & Li, T. (2020). Salt stress and fluctuating light have separate effects on photosynthetic acclimation, but interactively affect biomass. *Plant, Cell & Environment*, *43*(9), 2192–2206. https://doi.org/10.1111/pce.13810

Zheng, B., Li, Y., Wu, Q., Zhao, W., Ren, T., Zhang, X., Li, G., Ning, T., & Zhang, Z. (2023). Maize ( *Zea mays* L.) planted at higher density utilizes dynamic light more efficiently. *Plant, Cell & Environment*, *46*(11), 3305–3322. https://doi.org/10.1111/pce.14673

Zheng, Y., Blom, T., & Dixon, M. (2006). Moving lamps increase leaf photosynthetic capacity but not the growth of potted gerbera. *Scientia Horticulturae*, *107*(4), 380–385. https://doi.org/10.1016/j.scienta.2005.09.004

Zhu, X. G., Wang, Y., Ort, D. R., & Long, S. P. (2013). e-photosynthesis: a comprehensive dynamic mechanistic model of C3 photosynthesis: From light capture to sucrose synthesis. *Plant, Cell and Environment*, *36*(9), 1711–1727. https://doi.org/10.1111/pce.12025
